# Inactivity and the passive slowing effect of cold on resting metabolism as the primary drivers of energy savings in overwintering fishes

**DOI:** 10.1242/jeb.243407

**Published:** 2022-04-21

**Authors:** Connor Reeve, Lauren E. Rowsey, Ben Speers-Roesch

**Affiliations:** Department of Biological Sciences, University of New Brunswick, Saint John, New Brunswick, Canada, E2L 4L5

**Keywords:** Metabolic rate, Energy expenditure, Activity, Temperature, Fish, Hibernation, Torpor

## Abstract

Winter dormancy is a seasonal survival strategy common among temperate ectotherms, characterized by inactivity, fasting and low metabolic rates. Previous reports of metabolic rate depression (MRD) in winter-dormant ectotherms, including many fishes, may have resulted from confounding influences of temperature-dependent variation in activity on metabolic rate measurements. We hypothesize that, as demonstrated recently in the winter-dormant cunner (*Tautogolabrus adspersus*), inactivity and the passive physicochemical (Arrhenius) effect of cold on standard metabolic rate (SMR) are the common primary mechanisms underlying the low metabolic rates among winter-dormant fishes. Using automated video tracking, we investigated threshold temperatures for winter dormancy onset (major reductions in activity, increased sheltering and fasting) in four phylogenetically diverse teleost species reported to be winter dormant: cunner, pumpkinseed sunfish (*Lepomis gibbosus*), American eel (*Anguilla rostrata*) and mummichog (*Fundulus heteroclitus*). All species showed large activity and feeding reductions, but the magnitude of change and dormancy threshold temperature was species-specific. We propose that a continuum of overwintering responses exists among fishes from dormant to lethargic to active. The relationship between activity and metabolic rate was then measured using video-recorded automated respirometry during acute cooling and following cold acclimation in pumpkinseed, mummichog and eel. In all species, activity and metabolic rate were strongly correlated at all temperatures, and cooling caused reduced activity and metabolic rate. When variation in activity was controlled for across temperatures spanning the dormancy thresholds, the thermal sensitivity of metabolic rate including SMR indicated the predominance of passive physicochemical influences (mean *Q*_10_<3.5), rather than active MRD. Activity reductions and physicochemical slowing of metabolism owing to cold appear to be the primary energy-saving mechanisms in overwintering fishes.

## Abstract (precis)

**Highlighted Article:** Overwintering behaviour among fishes ranges from dormant to lethargic to active. Energy savings come from activity reductions and passive slowing of metabolism by the cold, not from metabolic rate depression.

## INTRODUCTION

Dormancy is a common strategy among animals to cope with the challenging cold and energy-limited winters at temperate to polar latitudes (McNab, 2002). Winter dormancy is a reversible seasonal phenotype characterized by inactivity, low body temperature, fasting and a low metabolic rate (McNab, 2002; Shuter et al., 2012; Boyles et al., 2013; Speers-Roesch et al., 2018). Winter dormancy may be a useful strategy to facilitate the persistence of a species at the cold limit of its range and could be viewed as a novel tactic to expand a species' poleward geographic range (Stuart-Smith et al., 2017; Speers-Roesch et al., 2018).

Many temperate fish species engage in dormancy during overwintering (Roberts, 1964; Nyman, 1972; Targett, 1978; Crawshaw et al., 1982; Walsh et al., 1983; Crawshaw, 1984; Lemons and Crawshaw, 1985; Sayer and Davenport, 1996; Raposa, 2003; Costa et al., 2013; Tomie et al., 2013; Speers-Roesch et al., 2018). For example, cunner, American eel and brown bullhead (*Ameiurus nebulosus*) have been reported to fast and remain buried within the substrate during winter (Green and Farwell, 1971; Nyman, 1972; Crawshaw et al., 1982; Walsh et al., 1983). Similarly, many centrarchid sunfishes markedly decrease their activity and feed little in the cold (Lemons and Crawshaw, 1985; Suski and Ridgway, 2009). However, few studies have directly measured and characterized the temperature-dependent behaviour of dormant fishes, especially in a comparative context.

The involvement of metabolic rate depression (MRD) in winter-dormant fishes is controversial. MRD is an active, reversible depression of resting cellular energy turnover that lowers an animal's whole-animal metabolic rate to well below their standard or basal (i.e. resting) metabolic rate (SMR). MRD is a common mechanism facilitating the persistence of animals during energy-limited periods (Storey and Storey, 2004). Winter dormancy in ectotherms is often considered analogous to mammalian hibernation, where profound MRD is common (Crawshaw, 1984; Costa et al., 2013). However, excluding species that overwinter in anoxic waters (e.g. certain freshwater turtles; Staples, 2016), controversy exists over the involvement of MRD in the numerous winter-dormant ectotherms that overwinter under normoxic conditions (Ultsch, 1989; McNab, 2002). This is partially due to the difficulty of distinguishing MRD from lethargy and slowed metabolism at cold temperatures (McNab, 2002).

The involvement of MRD in winter-dormant ectotherms can be assessed using the thermal sensitivity quotient (*Q*_10_) of metabolic rate (specifically, SMR) over the transition from an active to a dormant state (Speers-Roesch et al., 2018). The effect of temperature on metabolic rate in ectotherms, including fish, is relatively conserved across taxa and is commonly associated with a *Q*_10_≈2–3 (Peck, 2016; Clarke, 2017), which reflects the direct, passive physicochemical (i.e. Arrhenius) effects of temperature on underlying cellular biochemistry. At low temperatures, typical Arrhenius *Q*_10_ values for metabolic rate tend to be slightly higher compared with warmer temperatures (Clarke, 2017). Therefore, conservatively, a cold-induced decrease in metabolic rate with a *Q*_10_>3.5 has commonly been taken to indicate MRD, where the animal is actively suppressing its metabolic rate to a greater extent than would be predicted to arise from passive thermal effects on metabolism alone (Crawshaw, 1984; Costa et al., 2013; Staples, 2016; Speers-Roesch et al., 2018).

Using this approach, previous studies have argued both for (Roberts, 1964; Targett, 1978; Walsh et al., 1983; Sayer and Davenport, 1996; Costa et al., 2013) and against (Crawshaw et al., 1982; Lemons and Crawshaw, 1985; Costa et al., 2013; Speers-Roesch et al., 2018) the presence of MRD in various winter-dormant fish species, suggesting considerable interspecific variation in the capacity for MRD. For example, studies on winter-dormant brown bullhead (*Ameiurus nebulosus*) and largemouth bass (*Micropterus salmoides*) reported typical physicochemical effects of temperature on metabolic rate (*Q*_10_≈2–3) associated with inactivity and lethargy, suggesting that dormancy may simply be a phase of inactivity where the fish remains at SMR (Crawshaw et al., 1982; Crawshaw, 1984; Lemons and Crawshaw, 1985). Alternatively, certain studies on winter-dormant American eel and temperate wrasses (Labridae) have reported disproportionately large decreases in metabolic rate with cooling to winter temperatures (*Q*_10_ values of 4.1 and 7.9–10.4+), which was taken as evidence for involvement of MRD (Walsh et al., 1983; Sayer and Davenport, 1996; Costa et al., 2013).

Interpretation of these previous studies is complicated because many of them did not account for the potential influences of temperature-dependent variation in activity on metabolic rate, which can confound estimates of SMR (i.e. resting metabolic rate). MRD is a depression of metabolic rate below SMR, so identification of MRD in winter dormancy is dependent upon accurate measurements of SMR at each test temperature. However, SMR can be difficult to measure because of the challenge of ensuring that metabolic rate measurements are always made on resting fish (Chabot et al., 2016). Ideally, simultaneous measurement of activity alongside metabolic rate can be carried out to ensure that SMR is ascertained when fish are resting, yet this is rarely done (Chabot et al., 2016). Given that winter dormancy is characterized by inactivity, and spontaneous activity can cause substantial elevations of metabolic rate above SMR in fishes (Nilsson et al., 1993), reductions in activity in the cold could explain the large decreases in metabolic rates observed during dormancy.

In fact, a recent study demonstrated that when variation in spontaneous activity is controlled for in cunner, a winter-dormant species previously described to engage in MRD, the decrease in metabolic rate during winter dormancy was explained by the physicochemical effects of cold alone (*Q*_10_≈3) (Speers-Roesch et al., 2018). An outstanding question is whether these results are specific to cunner. We hypothesize that diminished activity, combined with passive cooling effects on SMR, is the primary, common mechanism underlying the low metabolic rate of winter-dormant fishes. To test this hypothesis, we first identified the dormancy threshold temperature (i.e. temperature of onset of inactivity, feeding cessation and increased sheltering) in four species of temperate fishes previously reported to be winter-dormant and of a broad phylogenetic range (cunner, pumpkinseed, mummichog and American eel), using automated video tracking of fish acutely cooled from summer to winter temperatures. We then investigated the relationship between spontaneous activity and metabolic rate during acute cooling and long-term acclimation to winter low temperatures in pumpkinseed, mummichog and eel to obtain accurate estimates of SMR and determine whether MRD or inactivity and the cold explains the low metabolic rates of winter-dormant fishes. We predicted that, in all species, activity would decrease while SMR *Q*_10_ would remain <3.5 (i.e. no MRD involvement) with cooling below the dormancy threshold and even after cold acclimation.

## MATERIALS AND METHODS

### Experimental species

Mummichog [*Fundulus heteroclitus* (Linnaeus 1766), family Fundulidae] adults of mixed sexes were collected from Sam Orr Pond, Bocabec, New Brunswick, in autumn 2017 using a combination of minnow traps and seining. Pumpkinseed sunfish [*Lepomis gibbosus* (Linnaeus 1758), family Centrarchidae] adults of mixed sexes were collected in autumn 2018 from Lily Lake, Saint John, New Brunswick, using a combination of trap netting and seining. American eel [*Anguilla rostrata* (Lesueur 1817), family Anguillidae] juveniles were supplied by Atlantic Canada Eels Inc. in May 2018 and were wild-caught elvers returning to freshwater from the ocean. Cunner [*Tautogolabrus adspersus* (Walbaum 1792), family Labridae] juveniles were obtained from a Cooke Aquaculture captive breeding program at the Huntsman Marine Science Centre (HMSC) in January 2018 (F1 offspring of wild-caught parents reared in 2017, stock origin: Saint Mary's Bay, Nova Scotia).

Fishes were maintained for a minimum of 4 weeks prior to experimentation in holding tanks supplied with flow-through dechlorinated freshwater (pumpkinseed and American eel) or recirculating filtered seawater (mummichog and cunner) at the University of New Brunswick, Saint John (UNBSJ). The holding tanks contained numerous PVC pipe shelters. All species were fed every other day with dry pellets (1.5 mm Gemma, Skretting, St Andrews, New Brunswick, Canada) or, for American eel, bloodworms (San Francisco Bay Brand, Newark, CA, USA). The holding acclimation water temperature was a typical summer temperature of 14°C±0.6°C for cunner, mummichog and pumpkinseed sunfish, or 17°C±0.6°C for American eel. A higher holding temperature was used for eel because wild American eel seem to enter dormancy at a relatively warm temperature (Nyman, 1972; Riley et al., 2011; Westerberg and Sjöberg, 2015).

All species were maintained under a winter photoperiod (10 h:14 h light:dark), because it occurs in New Brunswick and Nova Scotia when water temperatures are cooling in the autumn (October) and fish are presumably preparing to enter winter dormancy, and in the middle of the winter when water temperatures are lowest and fish would be in winter dormancy (February). The diel light cycle included a simulated sunrise and sunset (30 min each) to minimize potential biological effects of sudden light changes (Ryu et al., 2020). All experiments were carried out under the same lighting conditions (10 h:14 h light:dark photoperiod with simulated sunrise and sunset).

Fish collections were approved by DFO Canada and experimental work was approved by the Animal Care Committee of UNBSJ, following the standards and guidelines outlined by the Canadian Council on Animal Care.

### Experiment 1: behavioural responses to cooling to winter temperature

In order to characterize behavioural responses to cold and to identify dormancy threshold temperatures, we used continuous video recordings and automated tracking software to measure spontaneous activity, food consumption and sheltering behaviour in each species during acute cooling (1°C day^−1^) from their initial warm acclimation temperature to a winter low temperature. Owing to the elongate shape, undulating swimming and small size of American eels, their behaviour could not be automatically tracked. Instead, an observer manually scored their ‘vigilance’, an assessment of their activity out of the shelter as well as their alertness within the shelter (see below).

The system to measure behaviour consisted of a clear acrylic aquarium (101×68×15 cm) standing on upright clear acrylic pipes and continuously illuminated from below by four infrared lamps (940 nm) (Fig. S1A–C). A white translucent sheet of acrylic was placed directly underneath the acrylic aquarium to diffuse the infrared light, silhouetting fish within the system. Mounted above the aquarium was an infrared-sensitive digital video recording system (two cameras, each with 640×480 pixels, 10–15 frames s^−1^; IDS Imaging, Obersulm, Germany) that enabled daytime and nighttime video recordings of fish behaviour. Our study species appeared to be insensitive to infrared light, based on a lack of behavioural response to the turning on/off of infrared lamps (in contrast to an obvious startle response to the on/off of visible overhead lights), which is consistent with observations by Speers-Roesch et al. (2018).

Within the acrylic aquarium were plastic arenas (6 for pumpkinseed sunfish, 12 for American eel or 16 for cunner and mummichog), each for an individual fish and each fitted with a section of PVC pipe for shelter (Fig. S1A–C). The arenas were plastic boxes matched to species size to allow for sufficient room for exploratory behaviour; the shelters were sized to match typical species length and height (Table S1). The arenas were individually plumbed with tubing carrying water from a seawater or freshwater recirculating system (depending on the species) initially maintained at the species-specific holding acclimation temperature using a commercial water chiller (1/3 horsepower Arctica, JBJ Chillers, St Charles, MO, USA). Each arena had overflow holes that drained to the outer acrylic tank, and this in turn drained to a sump from which water was recirculated to the arenas following filtration and chilling.

**Fig. 1.**
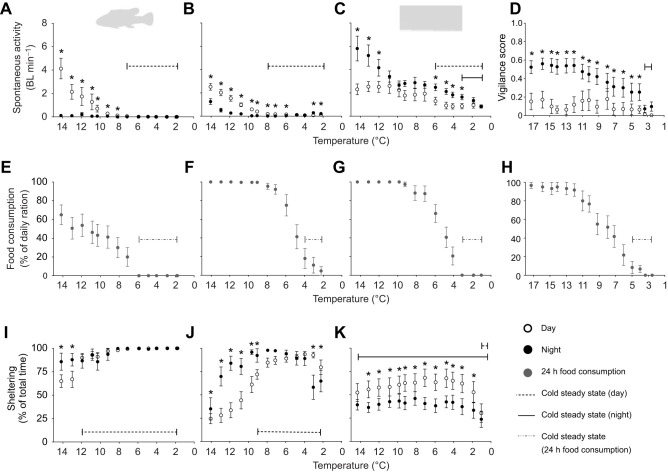
**Effects of acute cooling (1°C day^−1^) on activity-related behaviours and food consumption in four species of putatively winter-dormant fishes (experiment 1).** The species are arranged vertically (cunner, *n*=16, A,E,I; pumpkinseed sunfish, *n*=12, B,F,J; mummichog, *n*=16, C,G,K; American eel, *n*=12, D,H), and behaviours are arranged horizontally (spontaneous activity, A–C; vigilance in eels, D; food consumption, E–H; sheltering, I–K). Data are means±s.e.m. Open and closed circles represent daytime and nighttime measurements, respectively. Food consumption was measured as the percentage of the daily ration (∼0.5% body mass) consumed over 24 h (grey closed circles, E–H). Spontaneous activity is the average velocity in body lengths per minute (BL min^−1^) of fish over the full daytime or nighttime period at each temperature. See Materials and Methods for description of vigilance score in eels. Daytime or nighttime cold steady states (dashed or solid lines, respectively) indicate the temperature range where behaviour or feeding was not significantly different (*P*>0.05) from the value at the coldest temperature within the daytime or nighttime. Cold steady states were only analysed for daytime in the diurnal cunner and pumpkinseed, and nighttime in the nocturnal eel. Mummichog were not distinctly diurnal or nocturnal, so two steady states were calculated. * indicates a significant difference between daytime and nighttime values at a given temperature (*P*<0.0001) (generalized linear mixed-effects models with Bonferroni *post hoc* multiple comparisons tests; see Table S2 for outputs).

For cunner, mummichog and American eel, a single experimental trial was run with all individuals; however, owing to their larger size, pumpkinseed were measured in two separate, sequential trials to reach the final sample size (cunner, *n*=16; pumpkinseed sunfish, *n*=12; mummichog, *n*=16; American eel, *n*=12). Fish were placed individually into the arenas and a sheet of clear acrylic was placed on top of all arenas to prevent fish escape. The experimental system was surrounded by black plastic bags to prevent disturbance and overhead lights in the lab were covered to minimize the potential effect of bright light on fish behaviour. The fish were given 2–4 days to become accustomed to their experimental arenas. The trial began with a 24-h (i.e. a complete light:dark cycle) measurement at the fish's respective acclimation temperature. The fish were then cooled every morning at approximately 08:30 h at a rate of ∼1°C day^−1^ from their acclimation temperature (14°C for mummichog, cunner and pumpkinseed sunfish; 17°C for American eel) to ∼2°C for cunner, pumpkinseed sunfish and American eel, and ∼1°C for mummichog. The daily 1°C cooling took approximately 30 min, during which time we carried out feeding counts for the previous day (see below). Therefore, the fish were exposed to each temperature for approximately 24 h. At each temperature, the behavioural parameters were measured over the daytime and nighttime and were averaged to obtain a single daytime and nighttime measurement for each fish for activity and sheltering (or vigilance in eels), and a single daily food consumption measurement (e.g. see Fig. 1) (see below for more details).

The temperature of the chilled water within the acrylic aquarium was recorded using a Traceable digital thermometer (Cole-Parmer Canada Company, Montreal, Quebec, Canada), which displayed temperature in real-time and also recorded the highest and lowest temperatures experienced during the 24-h period (i.e. representing the setpoint hysteresis of the chiller). An average daily temperature was calculated by averaging temperatures recorded in the morning (∼09:00 h), evening (∼17:00 h), and the highest and lowest temperatures recorded by the thermometer. The average daily temperatures during each species' acute cooling can be found in the supporting data in figshare (https://doi.org/10.6084/m9.figshare.19131593.v1) and were within ±0.2 to 0.4°C of the desired temperature.

During each trial, each fish was fed a ration of ∼0.5% body mass (BM) of dry pellets or blood worms (for American eels) every morning. Food consumption was determined at each temperature by counting the remaining pellets or worms collected 24 h later, before the next cooling step. Spontaneous activity and sheltering behaviour were calculated from daytime and nighttime video recordings using automated tracking software (ToxTrac, v2.84; Rodriguez et al., 2018). The first 1 h and last 1.5 h of the daytime and nighttime periods were removed from the measurement period to reduce the effect of disturbances (i.e. feeding and/or cleaning the lids of splashes or condensation to ensure fish remained visible) such that the daytime measurement period was 09:00–16:00 h and the nighttime measurement period was 19:00–06:00 h. The pixel-to-distance calibration necessary for appropriate calculations of distance moved was conducted using known distances and pixel measurements in ImageJ (version 1.52a, National Institutes of Health, Bethesda, MD, USA; Schneider et al., 2012). Our measurements of spontaneous activity represent the average speed of the fish over the daytime or nighttime period at each temperature, calculated as the total distance moved over that period (as measured by ToxTrac) divided by the total corresponding time and standardized to the fish's total length (average body lengths moved per minute, BL min^−1^). Sheltering behaviour was quantified as ToxTrac's calculated invisible time over the daytime or nighttime at each temperature, because invisible periods (i.e. periods where the software did not detect the fish) occurred when the fish were inside their opaque PVC shelter. Activity within the shelter was assumed to be zero because the shelter size was matched to the species size and individuals within a species were similar in size, thus providing minimal space for movement.

Vigilance was manually scored for American eels during daytime and nighttime at each temperature using the video recordings, following a modification of Nyman's (1972) behavioural scoring approach for eels. At every 30 min point, 1 min of video was visually assessed where each eel was scored using the following rubric: 1=fish out of shelter, 0.5=head out of the shelter, 0=fish fully enclosed within the shelter. These measurements were averaged for each eel across the daytime or nighttime at each temperature. American eel at normal temperatures are generally active at night, when they forage, but will spend their daytime (as well as winter) sheltering within burrows or in spaces among rocks or bottom debris (Nyman, 1972; Tomie et al., 2013). When sheltering at warmer active temperatures, eels often protrude their heads from their shelters to scan their environment, and this behaviour decreases with cooling (Nyman, 1972; authors’ personal observations). Thus, our method of vigilance scoring is suitable for assessing American eel activity.

### Experiment 2: effect of acute cooling and cold acclimation on spontaneous activity and metabolic rate, and their relationship

**Fig. 2.**
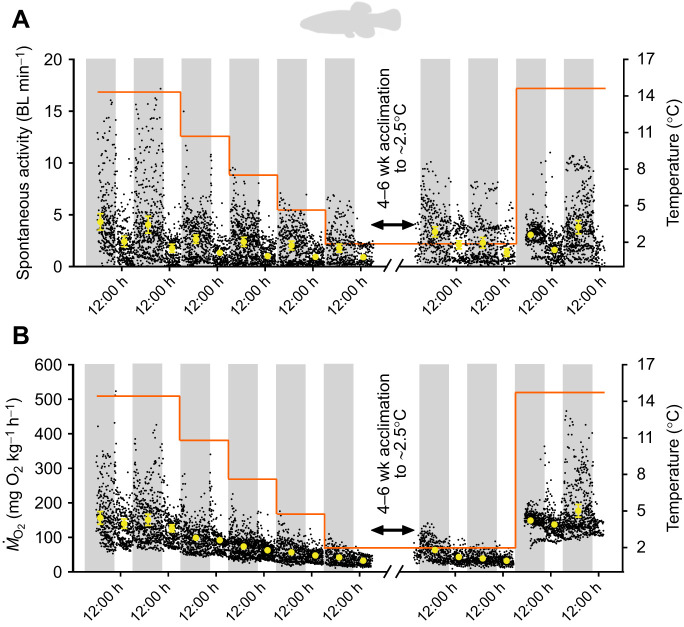
**Effects of acute cooling, cold acclimation and acute rewarming on spontaneous activity and metabolic rate (oxygen consumption rate, *Ṁ*_O_2__) of mummichog.** The diel cycle of activity (A) and *Ṁ*_O_2__ (B) was measured simultaneously during acute cooling (∼3°C day^−1^) and following 4–6 weeks acclimation to ∼2.5°C and acute rewarming to ∼14°C overnight (experiment 2). The black symbols are the spontaneous activity (A) and *Ṁ*_O_2__* *(B) values for all fish during all measurement intervals. The yellow symbols are the mean±s.e.m. values (*n*=12) for each daytime and nighttime period (represented by white and grey vertical bars, respectively) at each temperature. The orange line represents the experimental temperature regime. Spontaneous activity and *Ṁ*_O_2__ were significantly affected by temperature (χ^2^=103.806, d.f.=9, *P*<0.0001; χ^2^=989.295, d.f.=9, *P*<0.0001, respectively), diel cycle (χ^2^=82.071, d.f.=1, *P*<0.0001; χ^2^=19.370 d.f.=1, *P*<0.0001, respectively) and their interaction (χ^2^=54.916, d.f.=8, *P*<0.0001; χ^2^=24.747, d.f.=8, *P*<0.0017, respectively) (generalized linear mixed-effects models and Type II Wald chi-square tests, with Bonferroni *post hoc* multiple comparisons tests, *P*<0.05).

**Fig. 3.**
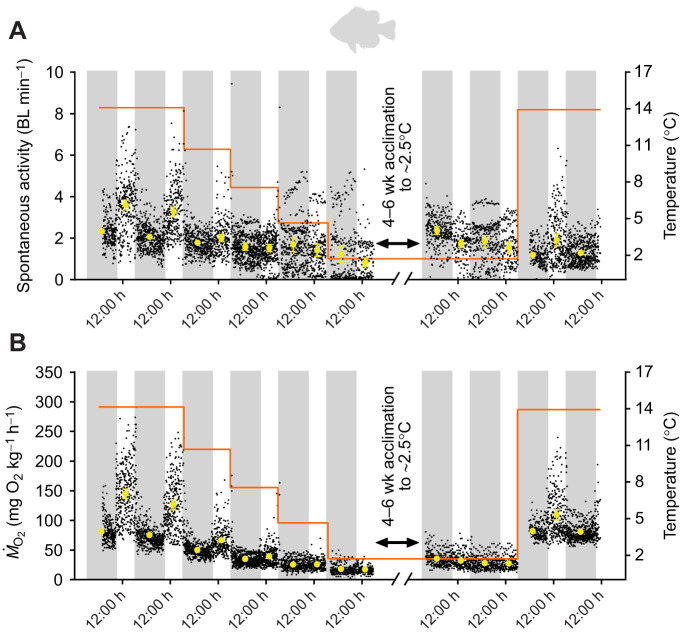
**Effects of acute cooling, cold acclimation and acute rewarming on spontaneous activity and metabolic rate (*Ṁ*_O_2__) of pumpkinseed sunfish (*n*=11).** The diel cycle of activity (A) and *Ṁ*_O_2__ (B) was measured simultaneously during acute cooling (∼3°C day^−1^) and following 4–6 weeks acclimation to ∼2.5°C and acute rewarming to ∼14°C overnight (experiment 2). Spontaneous activity and *Ṁ*_O_2__ were significantly affected by temperature (χ^2^=311.3361, d.f.=9, *P*<0.0001; χ^2^=1308.554, d.f.=9, *P*<0.0001, respectively) and their interaction between temperature and diel cycle (χ^2^=95.6660, d.f.=8, *P*<0.0001; χ^2^=17.619, d.f.=8, *P*<0.0243, respectively); however, only *Ṁ*_O_2__ was significantly affected by diel cycle (χ^2^=77.686, d.f.=1 *P*<0.0001). See Fig. 2 caption for further details.

**Fig. 4.**
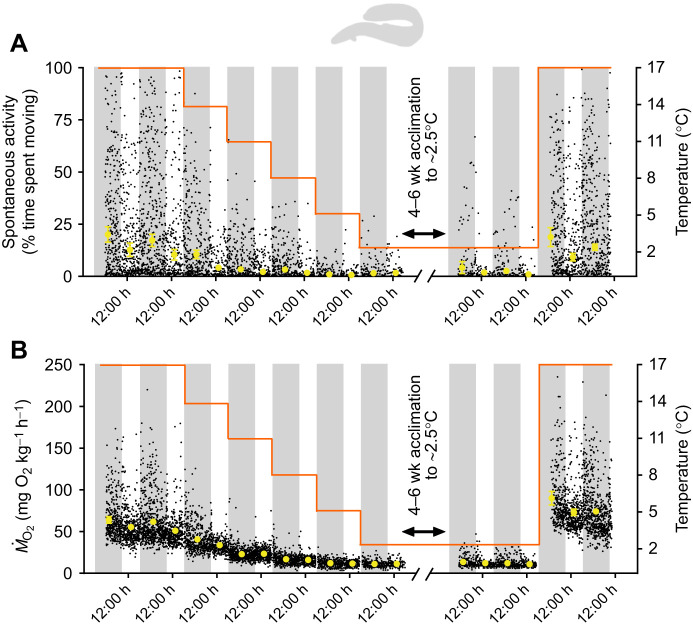
**Effects of acute cooling, cold acclimation and acute rewarming on spontaneous activity and metabolic rate (*Ṁ*_O_2__) of American eel (*n*=12).** The diel cycle of activity (A) and *Ṁ*_O_2__ (B) was measured simultaneously during acute cooling (∼3°C day^−1^) and following 4–6 weeks acclimation to ∼2.5°C and acute rewarming to ∼17°C overnight (experiment 2). Spontaneous activity and *Ṁ*_O_2_* *_were significantly affected by temperature (χ^2^=142.429, d.f.=10, *P*<0.0001; χ^2^=2518.715, d.f.=10, *P*<0.0001, respectively) and diel cycle (χ^2^=12.919, d.f.=1, *P*=0.0003; χ^2^=23.291, d.f.=1, *P*<0.0001, respectively). See Fig. 2 caption for further details.

We simultaneously measured metabolic rate and spontaneous activity in mummichog, pumpkinseed sunfish and American eel (see Table S1 for fish dimensions and sample sizes) during acute cooling (∼3°C day^−1^) from their initial warm acclimation temperature (∼15°C for mummichog, ∼14°C for pumpkinseed and ∼17°C for American eel) to ∼2.5°C, and after a subsequent 4–6 weeks of cold acclimation at ∼2.5°C followed by an acute rewarming period where the fish were warmed (over ∼6–8 h overnight) to their initial acclimation temperature (see Figs 2, 3 and 4; see below for further details). A faster cooling rate was used in experiment 2, relative to experiment 1 (1°C day^−1^), to minimize the fasting duration of the fish (they were not fed within the respirometers). Using the relationship between activity and metabolic rate at each acute or acclimation temperature, we estimated the thermal sensitivity of metabolic rate at known and comparable levels of activity, including extrapolated zero activity (i.e. SMR). This general approach enabled us to control for the influence of activity on metabolic rate and estimate the thermal sensitivity of SMR in order to ascertain whether MRD was involved in winter dormancy (i.e. *Q*_10_>3.5 for SMR), as described in further detail below.

#### Acclimation systems and experimental measurement system

The experimental fish were held in species-specific acclimation systems each consisting of three separate 75 litre glass aquaria (4 fish per aquarium, *n*=12) containing four PVC pipe shelters each. The three aquaria in each system were supplied with recirculating, temperature-controlled (Arctica chiller), filtered and aerated seawater (mummichogs) or freshwater (pumpkinseed, American eel). To enable repeated measurements on the same individual fish, mummichog and pumpkinseed were individually marked with visible implant elastomer tags (Northwest Marine Technology Inc., Anacortes, WA, USA). The American eel were too small for successful elastomer tagging, so they were individually housed in labelled 2 litre plastic containers within their acclimation system aquaria (a PVC pipe shelter was provided within each container). After tagging, fish were held in the acclimation system for at least 4 weeks at the initial warm acclimation temperatures and fed every other day, before the experiment began.

The metabolic rate and spontaneous activity of fish was measured in an experimental measurement system (Fig. S1D–F) consisting of individual respirometers (Table S1) that were placed in a clear acrylic water bath (the same as used in experiment 1, allowing for recording of fish activity within respirometers; see below) and initially maintained under the same water conditions as the acclimation system. The respirometers for mummichogs and pumpkinseeds were custom-built clear acrylic circular chambers with nylon barbed fittings for flush and recirculation of water, and sealed with a removable water-tight acrylic lid affixed using stainless steel nuts and bolts. The outer sides of each respirometer were covered with black plastic sheet to minimize visual disturbance and mimic shelter. The recirculation loop was fitted with two T-fittings topped with water-tight cable glands through which oxygen and temperature probes were introduced to sample the chamber water. The respirometers for eels were custom-built using glass food containers with a water-tight lid (Tot Glass Baby Blocks Food Storage Containers, OXO, New York, NY, USA); the lid was affixed with sealing cable glands for water-tight introduction of probes to sample the chamber water. The water bath was supplied with recirculated water that was temperature controlled using both an Arctica chiller (1/3 horsepower Arctica, JBJ Chillers) and an Arctic A25 refrigerated circulator (Thermo Scientific, Newington, NH, USA) to provide even more precise temperature control.

#### Experimental protocol

Four experimental trials were run per species with three fish per trial (*n*=12 fish per species). All 12 individuals of a given species were held in the same acclimation system and the start day of each trial group's measurement period was staggered, ensuring a consistent temperature exposure timescale. For each trial, there were two measurement periods during which metabolic rate and spontaneous activity were simultaneously recorded continuously during both daytime and nighttime: initial warm-acclimated followed by acute cooling, and cold-acclimated followed by acute rewarming (see below). For each measurement period, three fish were transferred into the individual respirometers within the experimental measurement system. Fish were always fasted for 48–72 h prior to transfer. The fish were allowed to recover in the respirometer overnight for ∼8–10 h, after which the fish had returned to a stable metabolic rate, and the measurement period began.

For each experimental trial, the fish were initially measured at their warm acclimation temperature for 2 days (i.e. ∼15°C for mummichog, ∼14°C for pumpkinseed sunfish and ∼17°C for American eel), following which they were acutely cooled at a stepwise rate of ∼3°C day^−1^ until the fish reached ∼2.5°C. The cooling was initiated at ∼17:30 h every day and took between 15 and 60 min to cool (depending on the temperature; i.e. it took longer for the system to reach colder set points). Thus, nearly 24 h of metabolic rate and spontaneous activity was measured at each acute temperature exposure. Following this acute cooling exposure, the fish were transferred back to their acclimation systems, which were pre-cooled to a winter low temperature of ∼2.5°C. The fish were then acclimated to ∼2.5°C for a period of 4–6 weeks and provided food every second day (3–4 times per week), though there was little to no food consumption. Following this cold acclimation period, the same subset of three fish were transferred back to the same respirometers for repeated measurements of metabolic rate and spontaneous activity at ∼2.5°C for 2 days. While still in the respirometer, the fish were then acutely warmed from ∼2.5°C to their initial warm acclimation temperatures over ∼6–8 h and, once the temperature had stabilized, recordings of metabolic rate and spontaneous activity continued for one full daytime–nighttime cycle. A summary of the experimental timeline and temperature exposure regime for each species is illustrated alongside their measurements of metabolic rate and spontaneous activity in Figs 2, 3 and 4.

#### Measurement of metabolic rate and spontaneous activity

Metabolic rate was estimated by measuring oxygen consumption rate (*Ṁ*_O_2__, mg O_2_ kg^−1^ h^−1^) using automated intermittent-closed optical respirometry. Each respirometer was fitted with an individual optode and temperature probe to measure the within-chamber temperature-compensated oxygen level, using a four-channel FireSting with a four-temperature extension module (PyroScience, Aachen, Germany). The respirometer water was mixed by recirculation through an Eheim water pump for mummichog and pumpkinseed (Eheim 300, 5 l min^−1^, clamped to a low flow with plastic screw clamps turned a specific number of rotations to achieve a consistent flow), or for eels with stir-bars separated from the fish by plastic mesh and driven by submersible stirrers beneath each respirometer (Telemodul 20C with micro stirrers, Thermo Scientific). The closed and flush periods were modified depending on water temperature and fish:respirometer volume (Table S1). *Ṁ*_O_2__ was measured from the slope of the decline in water oxygen content during the closed period; the slopes were extracted using LabChart (version 8.1.13, ADInstruments, Colorado Springs, CO, USA). The first 5 min of each closed period (i.e. the equilibration period following flush) was excluded from slope calculation. A blank respirometer containing no fish was run simultaneously alongside the three fish in every measurement period to correct for background respiration.

To measure spontaneous activity simultaneously with metabolic rate, the respirometers were illuminated from either below (mummichog and pumpkinseed sunfish) or from the side (American eel; owing to use of magnetic stirrers underneath respirometers) with infrared lights (940 nm) and video recorded with infrared-sensitive cameras, as described previously for experiment 1. For each interval of time where metabolic rate was calculated for a given fish, the associated spontaneous activity was also calculated from the video of the fish within the respirometer over the same interval. For mummichog and pumpkinseed sunfish, spontaneous activity was calculated using ToxTrac as described for experiment 1. ToxTrac is sensitive enough to track minor postural adjustments or the fish being slightly buffeted by the recirculating water flow, neither of which is active spontaneous movement of the fish itself. Thus, we removed these effects for each fish by calculating individual ‘inactive control values’. These inactive control values were calculated by measuring each individual fish's movement in ToxTrac over three periods (∼10 min each) of known inactivity (i.e. visually assessed from video) and were subtracted from all of the measurements of spontaneous activity for each fish, thus removing the influence of minor non-activity movements (mummichog corrections averaged 0.54±0.03 BL min^−1^, *n*=12 and pumpkinseed corrections averaged 0.47±0.02 BL min^−1^, *n*=11). Because the stir-bars required for respirometry of American eels interfered with automated tracking, eel spontaneous activity could not be measured using ToxTrac. Instead, American eel spontaneous activity was measured by manually recording the time spent moving (Speers-Roesch et al., 2018) for each individual eel, so spontaneous activity in eels is reported as the percentage of the total time spent moving within a metabolic rate measurement interval.

### Data analysis and statistics

For all analyses, statistical significance was accepted at *P*<0.05 and all values presented in the text are means±s.e.m., unless otherwise noted.

#### Experiment 1: behavioural responses to cooling to winter temperature

Measurements of daytime and nighttime behaviour (i.e. spontaneous activity, sheltering, feeding and vigilance) were obtained for each fish at each temperature during the acute cooling trial. The effects of acute cooling on behaviours during daytime and nighttime were assessed using generalized linear mixed-effects models (GLMM; family=Gamma, link=inverse) in R (Version 3.5.1) (https://www.r-project.org/) using the function glmer (lme4 package; Bates et al., 2015). To assess changes in spontaneous activity and sheltering behaviour in response to cooling as well as diel period (daytime versus nighttime), GLMMs (family=Gamma, link=inverse) were run using individual spontaneous activity or sheltering data related to experimental temperature in combination with daytime and nighttime periods, with fish as a random factor nested within trial where necessary (i.e. multiple trials were not run for all species). The effect of acute cooling on feeding was determined using GLMMs of individual feeding data related to temperature with fish as a random factor nested within trial where necessary. In some instances, in order to fit the GLMMs to the data, rescaling of the data (e.g. dependent variable/1000) was performed. Significant effects were identified using the Anova function (car package; Fox and Weisberg, 2019) in R, which calculated *P*-values using Type II Wald chi-square tests. After fitting GLMMs to these various data, Bonferroni *post hoc* multiple comparisons tests were completed using the estimated marginal means (emmeans) package (https://CRAN.R-project.org/package=emmeans).

Each species-specific winter dormancy threshold temperature was defined by the temperature at which the fish reached a steady-state level of behaviour that was not significantly different from the measurement at the coldest temperature (e.g. ∼2°C). In other words, below the dormancy threshold temperature, the behavioural measurements, in response to further cooling, would not differ significantly. For this analysis, we only examined each species' data for the period of the day (i.e. either daytime or nighttime) where it was most active under normal warm conditions (i.e. corresponding to each species' diurnal or nocturnal activity pattern). This was daytime for the diurnal cunner and pumpkinseed, nighttime for the nocturnal American eel, and both daytime and nighttime for mummichog as they showed marked activity at all times with no clear diel cycle of activity. This was done because species with diel cycles of activity are already relatively inactive in their resting period of the day. We also calculated mean inactive and mean fasting temperatures, if they existed, to further characterize the species-specific dormancy response. These temperatures are defined as the average temperature at which individual fish entered an inactive (zero activity) or fasting (zero feeding) state, respectively.

#### Experiment 2: effects of temperature on diel cycles of *Ṁ*_O_2__ and spontaneous activity

Measurements of *Ṁ*_O_2__ and spontaneous activity in each fish were averaged across all measurement intervals for that fish and we calculated an average daytime and nighttime value for *Ṁ*_O_2_ _and corresponding spontaneous activity for each fish at each experimental temperature. One pumpkinseed was removed from analysis owing to a malfunctioning oxygen probe. To determine whether a diel cycle existed for *Ṁ*_O_2__ and spontaneous activity and whether temperature had a significant effect on activity and its diel cycle, GLMMs (family=Gamma, link=inverse) were applied in R using the function glmer. Individual averaged *Ṁ*_O_2__ or spontaneous activity measurements were related to temperature in combination with daytime and nighttime periods with fish as a random factor nested within trial. Significant effects were determined by using the Anova function in R, which calculated *P*-values using Type II Wald chi-square tests. After fitting GLMMs to these various data, Bonferroni *post hoc* multiple comparisons tests were completed using the emmeans package.

#### Experiment 2: estimating thermal sensitivities (*Q*_10_) of SMR and metabolic rate at a standardized activity level

##### Controlling for the effects of variation in activity on metabolic rates

We used three approaches to control for the effect of variation in activity on metabolic rate during cooling and acclimation to winter cold, allowing us to obtain estimates of SMR (i.e. metabolic rate at zero activity) at each temperature as well as estimates of metabolic rate at a similar, standardized level of activity (‘activity-controlled metabolic rate’) at each temperature (see Fig. S2 for visualizations of each approach). The thermal sensitivity of SMR or activity-controlled metabolic rate was then determined using the *Q*_10_ equation (see below), to determine whether, when contributions of activity were removed or controlled for, there was evidence of an active depression of metabolic rate (i.e. *Q*_10_>3.5) or simply passive physicochemical effects of cooling (*Q*_10_≈2–3). Using three approaches to control for the effect of variation in activity on metabolic rate allowed us to robustly interrogate our data and corroborate our evidence for or against involvement of MRD. Additionally, to show the effect of not controlling for variation in activity on estimates of metabolic rate thermal sensitivity, we calculated routine metabolic rate (using all *Ṁ*_O_2__ values at each temperature) and its *Q*_10_ for each species (see Fig. S3, ‘*Q*_10 (Average *Ṁ*_O_2__)_’ in Table S3).

###### Approach 1: Estimating SMR of individual fish

The primary approach used to calculate SMR at each temperature in each species involved controlling for variation in spontaneous activity by correlating measurements of spontaneous activity with their corresponding *Ṁ*_O_2__ values for all measurement intervals for each individual fish at each experimental temperature (Prism 6, GraphPad Software, Inc., San Diego, CA, USA) (Fig. S2A). The resulting relationship was described using the exponential equation *y*=*a*e*^bx^*, where *a* is SMR (extrapolated *Ṁ*_O_2_* *_at zero spontaneous activity, i.e. the *y*-intercept), *b* is the slope, *y* is *Ṁ*_O_2__ and *x* is spontaneous activity (Brett, 1964; Korsmeyer et al., 2002). Thus, for each individual fish, several exponential regressions were generated to determine its SMR at each experimental temperature (i.e. ∼17°C for American eel only, ∼14°C, ∼11°C, ∼8°C, ∼5°C, ∼2.5°C, 4–6 week acclimated ∼2.5°C, and acutely warmed ∼14–17°C depending on the species). This relationship provides a robust method of making predictions beyond a measured range, which is particularly beneficial for estimating SMR by extrapolating *Ṁ*_O_2__ values to zero spontaneous activity (Korsmeyer et al., 2002).

Estimates of SMR in individual fish were secondarily calculated by averaging a subset of its lowest 20 *Ṁ*_O_2_* *_values at each temperature, or by averaging the *Ṁ*_O_2__* *values corresponding to a subset of the lowest 20 spontaneous activity points at each temperature. We calculated SMR using these secondary methods to assess whether less rigorous methods of SMR estimation, relative to the extrapolation method described above, would nevertheless provide a consistent result for SMR thermal sensitivity. Thus, we do not report these SMR values, but we do report their *Q*_10_ values in Table S3 (‘*Q*_10 (Lowest 20 *Ṁ*_O_2__)_’ and ‘*Q*_10 (Lowest 20 SA)_’).

All individual relationships between spontaneous activity and *Ṁ*_O_2_* *_in American eel were found to be significant (*P*<0.05); in pumpkinseed and mummichog, most relationships were significant (*P*<0.05), but a few failed to reach significance (typically because of a small spread in activity values). In these few instances, the extrapolated SMR values were replaced with values calculated by the aforementioned secondary methods of individual SMR calculation, and we observed if there was any effect on our estimate of average *Q*_10_. In all cases, swapping in these values had no effect on the average *Q*_10_ value (compare ‘*Q*_10 (Ind Extrapolated)_’, which are the values calculated using individual SMR from approach 1, with ‘*Q*_10 (Extrapolated+*Ṁ*_O_2__)_’ and ‘*Q*_10 (Extrapolated+SA)_’ in Table S3).

###### Approach 2: Estimating group SMR

To support the analysis using individual SMR estimates, a single group SMR value for each species at each temperature was calculated. This was done by calculating the extrapolated *Ṁ*_O_2__ at zero activity using the exponential regression *y*=*a*e*^bx^* (see above) and including all measurements of spontaneous activity and corresponding *Ṁ*_O_2__ across all individual fish of a species at each temperature (Fig. S2B).

###### Approach 3: Metabolic rate at a similar level of activity (activity-controlled metabolic rate)

In addition to estimating SMR, we calculated the *Ṁ*_O_2__ of individual fish at each temperature within a narrow, overlapping range of spontaneous activity that occurred at all temperatures (Fig. S2C). This approach controls for activity variation while avoiding any potential imprecision of the extrapolation of SMR values as done in approaches 1 and 2. Owing to differing levels of spontaneous activity measured in the pre- and post-acclimation trials in both mummichog and pumpkinseed sunfish, different narrow overlapping ranges had to be used to calculate the thermal sensitivity of acute cooling (between 0.8 and 1.8 BL min^−1^ for both mummichog and pumpkinseed sunfish) and acute rewarming (between 2.0–2.5 and 1.0–1.5 BL min^−1^ for mummichog and pumpkinseed sunfish, respectively), and the thermal sensitivity between ∼14–15°C acclimated and ∼2.5°C acclimated mummichog and pumpkinseed could not be calculated. For eels, their overlapping range of spontaneous activity was between 0 and 5% of time spent moving. These values allowed for thermal sensitivity analysis of *Ṁ*_O_2__ values where the contribution of variation in activity across temperature has been controlled for.

##### Thermal sensitivity analysis of SMR and activity-controlled metabolic rate

The thermal sensitivities of metabolic rate, including SMR and activity-controlled metabolic rates for individual fish and group SMR values, were calculated using the temperature (*T*) quotient (*Q*_10_): *Q*_10_=(*Ṁ*_O_2_cold_/*Ṁ*_O_2_warm_)^[10/(*T*_cold_−*T*_warm_)]^. *Q*_10_ values were calculated for several relevant temperature intervals. During acute cooling, *Q*_10_ was calculated for the full temperature interval (i.e. warmest to coldest) as well as the warm and cold halves of the thermal change, which generally bracketed the temperature of onset of dormancy behaviours (i.e. ∼14°C to ∼2.5°C, ∼14°C to ∼8°C, ∼8°C to ∼2.5°C). *Q*_10_ was also calculated for the acute rewarming (i.e. 4–6 week acclimated 2.5°C to acutely warmed 14°C), for comparison with the acute cooling *Q*_10_. Finally, *Q*_10_ was calculated for the 4–6 week acclimation to winter low temperature (i.e. ∼14°C-acclimated to ∼2.5°C-acclimated). Using these temperature intervals for *Q*_10_ calculation enabled us to elucidate whether MRD, if present, occurred acutely in fish or if the onset of MRD was delayed (i.e. following acclimation). Alternatively, the comparison of ∼14°C and ∼2.5°C acclimated animals could indicate a decrease in thermal sensitivity, suggesting compensation following cold acclimation. All *Q*_10_ values are displayed in Table S3; please see caption of Table S3 for a detailed summary. To corroborate our *Q*_10_ analysis, we generated Arrhenius plots for mean SMR during acute cooling only (1/*T* versus lnSMR), and used the segmented function in R to identify breakpoints in thermal sensitivity.

##### Statistical analysis of metabolic rates and their thermal sensitivity

The effect of temperature on SMR in mummichog, pumpkinseed sunfish and American eel was tested with a linear mixed-effects model (LMM) using the lmer function in R that included repeated measures (lme4 package; Bates et al., 2015). In some instances, in order to fit the LMM to the data, rescaling of the data (e.g. dependent variable/1000) was performed. Significant effects were identified using the Anova function in R, which calculated *P*-values using Type II Wald chi-square tests. After fitting LMMs to these data, Bonferroni *post hoc* multiple comparisons tests were completed using the emmeans package. The same test was used to determine whether *Q*­_10_ was similar or different among the various temperature intervals identified above.

## RESULTS

### Experiment 1: Behavioural responses to cooling to winter temperature

At warm temperatures, cunner and pumpkinseed were more diurnally active, whereas eel were nocturnal, and mummichogs were active daytime and nighttime with higher activity generally occurring at nighttime (*P*<0.0001) (Fig. 1). In all species, cooling caused a reduction in activity, which greatly dampened the diel activity cycle (Fig. 1). This dampening is highlighted by a significant interaction of diel period and temperature on the spontaneous activity of cunner and pumpkinseed, and the vigilance of American eel (*P*<0.001). No significant interaction was observed in mummichog.

The cold-induced reduction in activity was greatest in cunner and pumpkinseed, where zero or near zero activity was recorded at and below 7.1°C and 7.9°C, respectively (Fig. 1A,B). Eel also exhibited near zero activity (using vigilance as a proxy) at the two coldest temperatures (3.0°C and 2.5°C), whereas mummichogs remained comparatively active even at the coldest temperature (1.1°C), albeit at a reduced level (Fig. 1C,D). Acute cooling resulted in decreased food consumption in all species (*P*<0.0001; Fig. 1E–H). All cunner, mummichog and eel ceased feeding at 5.9°C, 3.1°C and 3.4°C, respectively, and by 2.2°C all but one pumpkinseed had ceased feeding. Consistent with the observed reductions in activity, sheltering increased with cooling in cunner and pumpkinseed; however, no change in sheltering occurred in mummichog (Fig. 1I–K). Though not directly measured, American eel also showed increased sheltering, which contributed to their greatly reduced vigilance score (see Materials and Methods). Cunner showed the most profound sheltering response, spending nearly 100% of their time within their shelter at 8.2°C and below. Although pumpkinseed also spent more time sheltering with cooling, there was a small decrease in sheltering at the coldest temperatures (3.0°C and 2.2°C).

Species-specific dormancy threshold temperatures were identified by observing the cold steady states for each behavioural metric (i.e. the range of temperatures over which there was no significant change compared with the value at the coldest temperature). Comparable fasting and inactive (or reduced activity) steady states were observed in cunner (inactive steady state ≤7.1°C; fasting steady state ≤5.9°C), American eel (inactive steady state ≤3.4°C, fasting steady state ≤5.0°C) and mummichog, if focusing on their nocturnal activity (reduced activity steady state ≤3.1°C, fasting steady state ≤3.1°C). Pumpkinseed, however, exhibited more dissimilar inactive and fasting steady states (≤7.9°C and ≤4.0°C, respectively). To accompany these estimates of dormancy threshold temperatures, average inactive and average fasting temperatures were also calculated when inactivity and/or fasting was observed in a given species. These temperatures were generally similar to the steady-state temperatures in each species. Only cunner exhibited complete inactivity and fasting, which occurred on average at 7.3±0.6°C and 7.5±0.4°C. Mummichog and eel on average fasted at 4.4±0.3°C and 5.5±0.4°C, respectively, and pumpkinseed, as a population, did not fast or reach complete inactivity, although the levels were very low in the cold.

### Experiment 2: Effect of acute cooling and cold acclimation on spontaneous activity and metabolic rate

The spontaneous activity of mummichog, pumpkinseed and American eel in respirometers significantly decreased in response to acute cooling (*P*<0.0001; Figs 2–4). Diel cycles of activity were present in all species at warmer temperatures but were dampened at cooler temperatures, as supported by a significant interaction of diel period and temperature on the spontaneous activity of all species (*P*<0.05). At warmer temperatures, mummichog and American eel were nocturnal, with higher levels of activity during nighttime (*P*<0.05), whereas pumpkinseed were diurnal, with higher levels of daytime activity (*P*<0.05). Overall, these responses mirror those in experiment 1. Following 4–6 weeks of ∼2.5°C acclimation, spontaneous activity of mummichog and pumpkinseed in respirometers increased relative to the pre-acclimation values, suggesting some compensatory acclimation. This change was significant in pumpkinseed during both daytime and nighttime, but only significant for the daytime in mummichog (*P*<0.05). American eel spontaneous activity remained unchanged following cold acclimation. After acute re-warming to each species' initial holding temperature (i.e. ∼14–15°C for mummichog and pumpkinseed; ∼17°C for American eel), mummichog and pumpkinseed spontaneous activity did not change or decreased slightly, whereas American eel activity significantly increased to a level similar to their pre-cooling activity.

In all species, *Ṁ*_O_2__ (i.e. metabolic rate) closely tracked changes in activity (see below about their strong relationship). Diel cycles of *Ṁ*_O_2__ were observed at warmer temperatures but were dampened with cooling (i.e. significantly higher daytime or nighttime values were observed at the warmer but not colder temperatures; *P*<0.05) (see Figs 2–4), as supported by a significant interaction of diel period and temperature on the *Ṁ*_O_2__ of mummichog and pumpkinseed (*P*<0.05). *Ṁ*_O_2__ decreased in all species in response to acute cooling. Similar to their spontaneous activity, following 4–6 weeks of ∼2.5°C acclimation, *Ṁ*_O_2__ increased slightly in mummichog and pumpkinseed. This change was significant in pumpkinseed but was only significant for the first day of recording post-acclimation in mummichog (*P*<0.05). American eel *Ṁ*_O_2__* *remained unchanged following cold acclimation. Acute re-warming caused *Ṁ*_O_2__ in all species to increase significantly. In mummichog and pumpkinseed, *Ṁ*_O_2__ increased to a level similar to their pre-cooled *Ṁ*_O_2__, whereas American eel *Ṁ*_O_2_ _increased to a level significantly higher than their pre-cooled *Ṁ*_O_2__ (*P*<0.001).

### Experiment 2: Relationship between spontaneous activity and metabolic rate, and thermal sensitivity of SMR and activity-controlled metabolic rate

**Fig. 5.**
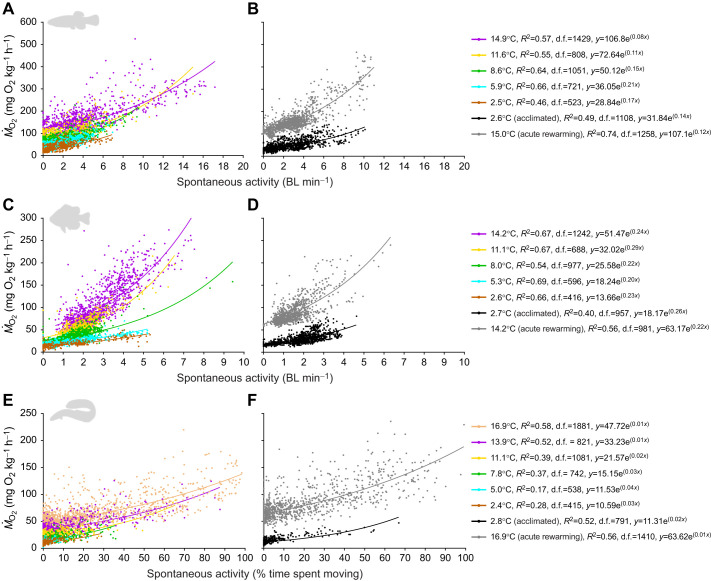
**Effects of acute cooling, cold acclimation and acute rewarming on the relationship between spontaneous activity and metabolic rate (*Ṁ*_O_2__) in three species of putatively winter-dormant fish.** The relationships are shown for mummichogs (A,B; *n*=12), pumpkinseed (C,D; *n*=11) and American eel (E,F; *n*=12) in all individuals at all measurement intervals within each experimental temperature (±0.3°C) during acute cooling (∼3°C day^−1^) (A,C,E) and after 4–6 weeks acclimation to ∼2.5°C followed by acute rewarming to the initial acclimation temperature overnight (B,D,F) (experiment 2). Within each temperature, the activity–*Ṁ*_O_2__ relationship was modelled exponentially: *y*=*a*e*^bx^*, where *y* is *Ṁ*_O_2__, *x* is spontaneous activity, *b* is the slope and *a* is SMR of the group (extrapolated *Ṁ*_O_2__ at zero spontaneous activity, i.e. the *y*-intercept). All relationships were significant (*P*<0.0001). See Table S3 for *Q*_10_ values of species-specific group SMR (‘*Q*_10_ Grp Extrapolated’). See Fig. 6 for individual-level SMR analysis.

Measurements of spontaneous activity and *Ṁ*_O_2__ were related exponentially to obtain estimates of group and individual SMR (i.e. extrapolated zero-point activity measurements of *Ṁ*_O_2__) for each species at each experimental temperature. There were strong significant relationships between spontaneous activity and *Ṁ*_O_2__ at each experimental temperature in all species when assessing each individual fish's values (i.e. approach 1, based on plotting all measurements of spontaneous activity and *Ṁ*_O_2_ _for an individual within a temperature) (individual relationships not shown), as well as for group values (i.e. approach 2, based on plotting all measurements of spontaneous activity and *Ṁ*_O_2__ across individuals within a temperature) (Fig. 5).

**Fig. 6.**
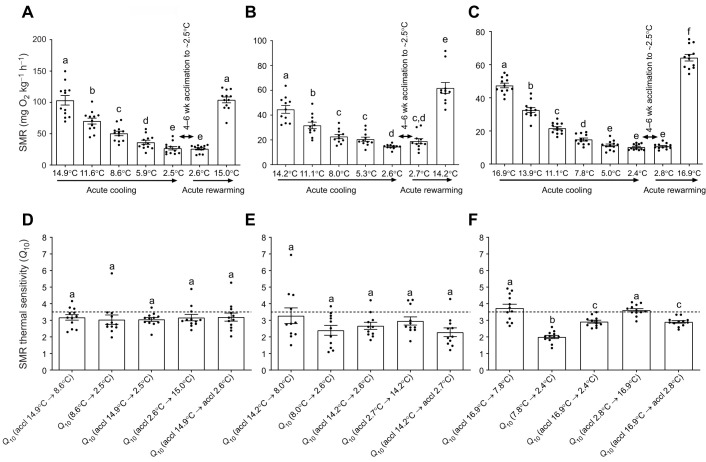
**Effects of acute cooling, cold acclimation and acute rewarming on the standard metabolic rates (SMR) and thermal sensitivities (*Q*_10_) of SMR in three species of putatively winter-dormant fish.** The SMR and *Q*_10_ of SMR are shown for mummichog (A,D), pumpkinseed (B,E) and American eel (C,F) during acute cooling from an initial warm acclimation (‘accl’) temperature, 4–6 weeks acclimation to cold (∼2.5°C) and acute rewarming of cold-acclimated fish (experiment 2). SMR were calculated by exponentially relating spontaneous activity versus metabolic rate in individual fish at a given temperature and extrapolating *Ṁ*_O_2_* *_at the *y*-intercept where activity is 0 (using *y*=*a*e*^bx^*, where *y* is *Ṁ*_O_2__, *x* is spontaneous activity, *b* is the slope and *a* is SMR of the individual; see Materials and Methods). *Q*_10_ values were calculated for individual fish using their SMR values shown in A–C across the specified temperature intervals (also reproduced as the ‘Ind Extrapolated’ *Q*_10_ values in Table S3). Data are means±s.e.m. (*n*=12, 11 and 12, for mummichog, pumpkinseed and American eel, respectively) with black circles representing the individual values. The dotted horizontal line represents our defined *Q*_10_ threshold inferring metabolic rate depression (i.e. *Q*_10_>3.5). Values with different letters are significantly different (linear mixed-effects models and Bonferroni *post hoc* multiple comparisons tests, *P*<0.05).

All species' SMR, calculated from the individual extrapolated zero-activity *Ṁ*_O_2__ values (approach 1), showed a significant decrease in response to acute cooling, no change following 4–6 weeks of ∼2.5°C acclimation, and a significant increase following acute re-warming to their initial pre-cooling temperature (Fig. 6A–C). In re-warmed mummichog, SMR returned to a level similar to their pre-cooling SMR, whereas in re-warmed pumpkinseed and American eel, SMR was significantly higher than their pre-cooling SMR (*P*<0.0001).

For mummichog and pumpkinseed, the thermal sensitivity of individual SMR was similar across all acute cooled, acclimated or acute rewarming temperature intervals, with a mean *Q*_10_ of <3.5 in all cases despite a few individuals with *Q*_10_ >3.5 (Fig. 6D,E). The thermal sensitivity of American eel SMR was more variable, but the mean *Q*_10_ was usually <3.5 (Fig. 6F). American eel had marginally higher mean *Q*_10_ values of 3.73 and 3.61 for SMR when acutely cooled from 16.9°C to 7.8°C and when acutely rewarmed from 2.8°C to 16.9°C (*P*<0.05) (Fig. 6F). Certain individual eels had SMR *Q*_10_ values above the 3.5 threshold (Fig. 6F). The individual SMR *Q*_10_ values (Fig. 6D–F) were similar to the *Q*_10_ values for group SMR (Fig. 5; approach 2; ‘*Q*_10_ Grp Extrapolated’ in Table S3) and the *Q*_10_ values calculated using each individual's *Ṁ*_O_2_* *_values within an overlapping activity range across temperatures (i.e. activity-controlled metabolic rate) (approach 3; ‘*Q*_10_ Overlapping SA’ in Table S3). Arrhenius plots of mean SMR during acute cooling corroborated our *Q*_10_ analysis, with no breakpoints except one in eels where higher thermal sensitivity occurred >6.3°C (C.R., unpublished observations/data not shown).

## DISCUSSION

Our findings support the hypothesis that inactivity, combined with the passive physicochemical slowing effect of cold on SMR, is the primary mechanism underlying energy savings in winter-dormant fishes, rather than MRD. In all of our study species, cooling caused persistent decreases in spontaneous activity, accompanied by reductions in *Ṁ*_O_2__ as well as feeding (Figs 1–4). The threshold temperatures of reductions and the magnitude of the activity reductions were species-specific (see below). However, at all temperatures, and in both acute and acclimation cold exposures, spontaneous activity was strongly positively correlated with metabolic rate in mummichog, pumpkinseed sunfish and American eel (Fig. 5), as found previously in cunner (Speers-Roesch et al., 2018). Spontaneous movements are an important contributor to metabolic rate in fishes, yet these are rarely measured simultaneously as we have done (Nilsson et al., 1993; Chabot et al., 2016). Therefore, reductions in activity with cooling accrued energy savings by decreasing locomotion costs and helping to lower metabolic rate closer to SMR. Cooling also caused SMR to slow in all species (Fig. 6), with thermal sensitivities that indicated the predominance of passive physicochemical effects on metabolism (*Q*_10_<3.5) (but see below regarding eels). The passive slowing of SMR thus accrued further energy savings, and there was no evidence of a disproportionately large decrease in SMR that would be expected if MRD was involved, even after several weeks of cold acclimation. A lack of MRD was corroborated by the observation of *Q*_10_ values <3.5 when calculating metabolic rates within a narrow overlapping activity range common to all temperatures (‘activity-controlled metabolic rate’), which controlled for temperature-dependent variation in activity. The conclusion of our multi-species analysis is consistent with the only previous studies where consideration was given to the potential influence of activity levels on metabolic rate in dormant fishes (Crawshaw, 1984; Crawshaw et al., 1982; Lemons and Crawshaw, 1985; Speers-Roesch et al., 2018). Overall, there is now strong evidence that energetic savings of winter dormancy in fishes result from inactivity and the passive physicochemical effects of cold on metabolism, not MRD.

Curiously, in American eel acutely cooled from 16.9°C to 7.8°C (over which they remain active and feeding; Fig. 1) and in response to acute warming from 2.8°C to 16.9°C, the average thermal sensitivities of SMR and activity-controlled metabolic rate was found to be marginally above the *Q*_10_=3.5 threshold for inferred MRD (*Q*_10_=3.51–3.73; Fig. 6F; Table S3). In contrast, the *Q*_10_ values were below the *Q*_10_=3.5 threshold for acute cooling from 7.8°C to 2.4°C and following 4–6 week ∼2.5°C acclimation, the temperature interval in which behavioural dormancy manifests (Fig. 1). These results provide some evidence of a modest active downregulation of SMR in American eel (possibly owing to downregulation of growth pathways, for example; Lewis and Driedzic, 2007; Lamarre et al., 2009), in anticipation of winter but decoupled from dormancy. However, for several reasons, we caution against interpreting this result as evidence of MRD per se. The calculated *Q*_10_ values were only marginally higher than our threshold, and much lower than the *Q*_10_ values typically observed where MRD has been (we think erroneously) inferred (e.g. *Q*_10_ values of 4.1–10.4+; Roberts, 1964; Targett, 1978; Walsh et al., 1983; Costa et al., 2013). Also, the individual fish *Q*_10_ values, and thus the variation around the mean, straddled the 3.5 threshold; some individuals had *Q*_10_ above 3.5 and some below, suggesting no consistent physiological response that would be expected from an adaptive MRD response. In fact, individual variation around the 3.5 *Q*_10_ threshold was also apparent in mummichog and pumpkinseed (though their average *Q*_10_ were <3.5). Possibly, some individuals have more thermally sensitive SMR simply because they are faster growers; with cold-induced slowing of growth (Clarke, 2017), they will show a greater decrease in SMR compared with a slow-growing individual. Although this hypothesis deserves attention, we argue this scenario does not reflect MRD, which is characterized by a comprehensive downregulation of energy demand pathways associated with the shift to a dormant phenotype (Staples, 2016). Indeed, eels continue feeding from 16.9°C to 7.8°C (Fig. 1). The >3.5 *Q*_10_ values following acute rewarming may have occurred because of a stress response resulting from the rapid warming (from 2.8°C to 16.9°C over ∼8–10 h), leading to elevated metabolic rate at rest. In contrast, acute cooling over the same temperature interval at the beginning of the experiment occurred more slowly (1°C day^−1^) and was associated with SMR *Q*_10_<3.5. Elevated SMR also occurred in pumpkinseed following acute rewarming when compared with SMR at their initial holding temperature (Fig. 6). In neither species does the elevated SMR upon rewarming reflect a compensatory cold acclimation of SMR, because SMR in the cold before and after the 4–6 week acclimation were similar, so a stress effect better explains the higher *Q*_10_ with rewarming. Further research is warranted on the significance and mechanisms of interindividual variation in thermal sensitivity of SMR, given the important role of SMR in setting the pace of life (Norin et al., 2016; Auer et al., 2018).

### The pattern and magnitude of winter-dormant behaviour is species-specific

To our knowledge, our study is the first to comprehensively quantify winter-dormant behaviour in a broad range of putatively winter-dormant fish species. Our study species have been previously reported to be winter-dormant (Roberts, 1964; Targett, 1978; Walsh et al., 1983; Sayer and Davenport, 1996; Speers-Roesch et al., 2018), so we predicted that large reductions in activity and feeding, accompanied by increases in sheltering, would be observed in all species at temperatures below species-specific ‘winter dormancy thresholds’. Winter dormancy threshold temperatures have been inferred for several dormant species based on anecdotal observations (Walsh et al., 1983; Costa et al., 2013; Westerberg and Sjöberg, 2015; Speers-Roesch et al., 2018); however, these thresholds are rarely, if ever, directly measured. Our experiment 1 demonstrates interspecific variation in winter dormant behaviour, with the most pronounced dormant behaviour occurring in cunner and the least in mummichog (Fig. 1).

#### Cunner

Cunner have a classic winter-dormant behavioural phenotype, where cooling causes pronounced inactivity, sheltering and fasting (Fig. 1). Our cunner, originating from southern Nova Scotia, became inactive at 7.3±0.6°C, and below 7.1°C all individuals were virtually inactive and sheltering, which is similar to previous reports of winter inactivity in wild or laboratory cunner in Newfoundland at <5°C (Green and Farwell, 1971; Bradbury and Green, 1997; Speers-Roesch et al., 2018). Our cunner also ceased feeding at 7.5±0.4°C, and none fed at ≤5.9°C. Fasting in winter-dormant cunner is known but has never before been quantified (Green and Farwell, 1971; Bradbury and Green, 1997; Speers-Roesch et al., 2018). Other long-term measurements on cunner in our lab show that persistent fasting or near-fasting and negative growth occurs in dormant cunner below 6°C (L.E.R., M. Watson and B. Speers-Roesch, unpublished observations). Overall, it appears that the cunner's dormancy threshold temperature ranges from 5 to 7°C, possibly dependent on latitude of origin.

#### Pumpkinseed sunfish

The behavioural response to acute cooling in pumpkinseed was similar to that of cunner, although the magnitude of change was less extreme. Pumpkinseed spontaneous activity decreased with cooling until, at 7.9°C, a minimally active steady state was reached, which is also similar to findings for other centrarchids after acclimation to <7°C (Lemons and Crawshaw, 1985; Tschantz et al., 2002). Like cunner, the large reduction in activity with cooling coincided with increased sheltering and a marked decrease in feeding below 7.9°C. However, pumpkinseed never reached inactivity, showed variable sheltering, and certain individuals continued to feed at a low level even at the coldest temperature. Little work has been done to quantify the overwintering sheltering or microhabitat selection in centrarchids (Suski and Ridgway, 2009), but our results are consistent with previous research showing greatly reduced (but not zero) activity and opportunistic winter feeding in pumpkinseed and other centrarchids (Collins and Hinch, 1993; VanderKooy et al., 2000; Tschantz et al., 2002; Block et al., 2020; Rooke and Fox, 2020). Although pumpkinseed appear to reach a minimally active steady state that may indicate the onset of dormancy at 7.9°C, the dormancy threshold is less clear for this species compared with cunner because certain individuals maintained a low level of activity and feeding in the cold. Therefore, the assignment of a dormancy threshold temperature for this species is challenging and may be unwarranted owing to the lack of completely developed dormant characteristics (i.e. inactivity and fasting) (see below).

#### Mummichog

Mummichog significantly reduced their spontaneous activity in response to acute cooling; however, unlike the other species tested, there was no clear cold steady state across the diel cycle, activity was persistent even at the coldest temperatures, and sheltering behaviour was relatively unaffected by cooling. The overwintering behaviour of mummichogs is poorly known, with various studies reporting any of a diverse range of strategies: migrating offshore into saltwater bays, migrating to saltier portions of creeks, migrating to less salty waters, migrating to tidal salt marsh pools, not migrating, or burying in substrate (Chidester, 1920; Fritz et al., 1975; Smith and Able, 1994; Haplin, 1997; Raposa, 2003). This variability led Fritz et al. (1975) to suggest that mummichog may be polytypic in their overwintering behaviour. Our mummichogs, from the Bay of Fundy, appeared to maintain a slow pace of activity in the cold (and show no evidence of burying in substrate; C.R., L. Vrooman and B.S.-R., unpublished observations). They also fed much less in the cold, like the other study species, with no feeding observed at ≤3.1°C (mean fasting temperature=4.4±0.3°C). Little is known about mummichog feeding during winter aside from anecdotes of gut contents (mostly algae; Chidester, 1920). Possibly, the fasting observed at the coldest temperatures in our study results from the acute temperature stress and, following acclimation, the mummichog would resume feeding. In fact, our unpublished observations (C.R., L. Vrooman and B.S.-R.) showed that although mummichog feeding decreased when acclimated for up to 4 weeks to 2–3°C, they sporadically fed at a low level. This is similar to pumpkinseed (although mummichogs maintain higher levels of activity), but different from the more dormant and fasting cunner and American eel (see below; L.E.R., M. Watson and B.S.-R., unpublished observations).

#### American eel

American eel showed decreased vigilance with cooling, indicating greater sheltering and reduced alertness at colder temperatures. In particular, a marked reduction in vigilance was observed at ≤3.4°C, with most eels remaining hidden within their shelters for the entire daytime and nighttime. This decrease in vigilance is consistent with previous studies on American eel and European eel (*Anguilla anguilla*) reporting inactivity and increased burying/sheltering within burrows during overwintering (Smith and Saunders, 1955; Nyman, 1972; Walsh et al., 1983; Tomie et al., 2013; Westerberg and Sjöberg, 2015). For example, Nyman (1972) found that European eel remained buried <8°C with no heads visible from burrows <6.4°C; American eel buried into mud at <5°C (Walsh et al., 1983). Greatly decreased activity and increased sheltering are common cold responses among anguillid eels.

The marked decrease in vigilance below 3.4°C in American eel coincided with fasting (Fig. 1), which is consistent with what little is known about winter feeding by eels. The stomach contents of overwintering European eel indicated fasting or minimal feeding (Sinha and Jones, 1967). American and European eels anecdotally fast below 5°C and 8°C, respectively (Nyman, 1972; Walsh et al., 1983). Overall, our measurements show that American eel engaged in minimal activity and all fasted at ≤3.4°C (mean fasting temperature=5.5±0.4°C), which, combined with previous literature on overwintering eels, is indicative of a dormant state (similar to that in cunner) below ∼3–4°C. The warmer threshold temperatures for sheltering and fasting behaviour noted in previous studies may result from interspecies, interpopulation, or methodological differences (Nyman, 1972; Walsh et al., 1983; Riley et al., 2011; Westerberg and Sjöberg, 2015).

#### The classification of overwintering strategies in fishes

Our comprehensive quantitative analysis of dormant behaviour among a range of phylogenetically diverse fish species indicates that winter dormancy behaviour is species-specific and varies in magnitude. A reappraisal of how we define winter dormancy and other overwintering responses in fishes is warranted, building on (but simplifying) Shuter et al.’s (2012) schema of winter survival strategies in fishes. We propose that overwintering strategies be classified along a spectrum as winter activity (winter-active), winter lethargy (winter-lethargic) or winter dormancy (winter-dormant), paralleling Shuter et al.’s (2012) designations of active, active-quiescent and quiescent winter survival strategies, respectively. Winter activity is defined by a relatively high level of activity, continued foraging (and growth) and little to no change in typical sheltering behaviour. Winter lethargy is an intermediate strategy defined by a marked reduction in activity, a low level of opportunistic feeding (which may sustain maintenance metabolism only, not growth; Block et al., 2020) and no specific sheltering behaviour (although it could involve moving to favourable overwintering habitat, e.g. a low water flow area). Winter dormancy is defined by inactivity, fasting (and negative growth) and sheltering. As shown in the present study, MRD is not a characteristic of overwintering in fishes. Our schema emphasizes quantitative phenotyping of behavioural and physiological traits to properly designate strategy to species and better reflect the continuity between overwintering strategies across species.

Our results, combined with previous findings, show that cunner and American eel are characteristic winter-dormant species; both cunner and American eel enter an inactive or virtually inactive state, and shelter and fast, at winter low temperatures (Sinha and Jones, 1967; Green and Farwell, 1971; Nyman, 1972; Walsh et al., 1983; Sayer and Davenport, 1996; Bradbury and Green, 1997; Riley et al., 2011; Westerberg and Sjöberg, 2015; Speers-Roesch et al., 2018). On the opposite end of the spectrum, winter activity is exemplified by yellow perch (*Perca flavescens*) and several *Salvelinus* and *Salmo* salmonid species, which can remain relatively active, foraging and growing throughout winter (Sullivan, 1986; Brännäs and Wiklund, 1992; Blanchfield et al., 2009; Amundsen and Knudsen, 2009; Block et al., 2020). Finally, pumpkinseed and mummichog are examples of winter-lethargic species, rather than winter dormant as previously reported, owing to the maintenance of some feeding and a low activity level at cold temperatures (present study; Block et al., 2020). Notably, winter lethargy encompasses a larger intermediate scope of activity, relative to winter dormancy or winter activity. For example, pumpkinseed showed greater decreases in activity and increases in sheltering compared with mummichog, consistent with a greater level of lethargy. Indeed, winter lethargy may be the most apt classification for many temperate fish species, especially centrarchid species, which have been described previously as winter-dormant despite evidence showing many of them engage in some activity and feeding, albeit with little or negative growth, during winter (Lemons and Crawshaw, 1985; Collins and Hinch, 1993; VanderKooy et al., 2000; Tschantz et al., 2002; Karchesky and Bennett, 2004; Suski and Ridgway, 2009; Block et al., 2020). The strategy used by any given species may correlate with its preferred temperature, with warm-preferring species tending to be lethargic or dormant in winter (Shuter et al., 2012), although this has not been explicitly evaluated. Also, intraspecific variation in overwintering strategies may occur in species that have a wide latitudinal range (Shuter et al., 2012), because of milder winters at the warm range limit, highlighting the continuum of overwintering strategies (e.g. more winter feeding and growth in low latitude largemouth bass; Garvey et al., 2004). We recommend that assessments of overwintering strategies in fishes involve precise and standardized behavioural phenotyping in the laboratory combined with field monitoring of winter activities, to facilitate quantitative investigations into the causes of variation in overwintering strategies among fishes.

### Activity reductions and the passive physicochemical effect of cold are primary drivers of energy savings in overwintering fishes

Our primary finding was that, as for cunner (Speers-Roesch et al., 2018), the low metabolic rates of mummichog, pumpkinseed sunfish, and American eel during winter dormancy did not result from MRD but rather from reduced activity in combination with the physicochemical effects of cooling on their metabolism. When SMR was estimated by extrapolating metabolic rate to zero activity (Korsmeyer et al., 2002), or when the influence of variation in activity on *Ṁ*_O_2__ was controlled for by using the average *Ṁ*_O_2__ over a narrow overlapping range of spontaneous activity, the thermal sensitivity (*Q*_10_) of SMR and activity-controlled metabolic rate in response to cooling below winter dormant temperatures in all species indicated the predominance of typical passive physicochemical effects alone (*Q*_10_<3.5) (Fig. 6, Table S3). The SMR of all species remained unchanged after 4–6 weeks acclimation at winter low temperature, contradicting the possibility of a delayed onset of MRD as seen in certain estivating amphibians (Hillman et al., 2009). However, neither was there a cold compensation (increase) of SMR following acclimation, mirroring previous findings for mummichog (Healy et al., 2017) and winter-dormant cunner (Speers-Roesch et al., 2018). Fish that minimize activity in winter may not benefit from thermal compensation of SMR, which has more typically been reported in active species (Peterson and Anderson, 1969; Evans, 1990). Our finding of ‘normal’ metabolic rate *Q*_10_ values when activity variation is controlled for matches Speers-Roesch et al.’s (2018) findings in cunner; previous reports of high thermal sensitivity of metabolic rate in cooled mummichog (*Q*_10_=4.42; Targett, 1978), pumpkinseed (*Q*_10_=6.0; Roberts, 1964) and American eel (*Q*_10_=4.10; Walsh et al., 1983) were likely confounded by unaccounted temperature-dependent variation in activity, rather than indicative of MRD.

Indeed, we found *Q*_10_ values were always higher (up to 5.6 in pumpkinseed) when comparing average *Ṁ*_O_2__ where variation in activity was not controlled for (i.e. routine metabolic rate; Fig. S3, Table S3). Essentially, if there is higher activity at warmer temperatures and lower activity at cold temperatures, this causes higher and lower metabolic rates to be recorded, respectively, inflating the difference between metabolic rates at the two temperatures and leading to erroneously high *Q*_10_ when activity is not accounted for. Relatedly, care should be taken when estimating SMR from a lowest subset of *Ṁ*_O_2__ measurements, a common procedure (Chabot et al., 2016); for example, we found that at warmer temperatures, mummichog and pumpkinseed were never inactive for an entire *Ṁ*_O_2__ measurement, which complicated SMR estimation from a lowest *Ṁ*_O_2__ subset and led to confoundedly higher *Q*_10_ values when compared with cold, less active animals (Table S3). We recommend simultaneous recording of activity with *Ṁ*_O_2__ whenever possible as the best method to accurately estimate SMR. Furthermore, to accurately calculate and interpret *Q*_10_, it is important to compare animals that are in similar, or at least controlled, behavioural states (Speers-Roesch et al., 2018; cf. Geiser, 2016). Accurate estimates of the relationship between activity and metabolic rate, and their responses to temperature, are vital to physiological and ecological theories that rely on the assumption of predictable universal thermal effects on metabolism among animals, and which are broadly applied to model the impacts of climate change (van der Meer, 2006; Dillon et al., 2010; Pörtner, 2010; Clarke, 2017).

A primary role for inactivity and passive thermal slowing of metabolic rate, rather than MRD, in driving energy savings in winter-dormant fishes was first suggested by Crawshaw and colleagues in largemouth bass and catfish (Crawshaw et al., 1982; Crawshaw, 1984; Lemons and Crawshaw, 1985), and later demonstrated by Speers-Roesch et al. (2018) in cunner. Here, we extend this conclusion to three additional fish species: mummichog, pumpkinseed sunfish and American eel. Importantly, despite the variation in overwintering behavioural responses seen among our study species (see above), all showed reductions in activity and exploited the slowing effect of cold, rather than compensating their metabolism via acclimation. Taken together, and considering the diverse phylogenetic range of fishes examined to date, the available evidence strongly indicates that reducing activity and allowing the cold to slow metabolism are the primary tools by which overwintering fishes save energy.

Compared with MRD, which does not appear to be used by overwintering fishes, activity reduction may be an optimal energy savings mechanism for ectotherms in cold environments. Suppression of activity is a simple behavioural response that obviates the evolution of more complex downregulation of metabolism via MRD, especially when paired with passive physicochemical slowing of metabolism in the cold. Exploitation of passive, yet still substantial, metabolic *Q*_10_ effects is important for overwinter survival in species that encounter hypoxia (Tattersall and Boutilier, 1997; Stecyk, 2017), but is also beneficial under normoxia given the unreliability of winter food. The activity reductions, in part, result from passive temperature effects on the physiology underlying movement (*Q*_10_=2–3; Shapley, 1924; Huey and Kingsolver, 2011), but also must represent an active behavioural response, as evidenced by: an associated increase in sheltering (in pumpkinseed, cunner and eel), very low activity in the cold despite our experience that the fishes could increase movement if disturbed, and high thermal sensitivities for activity in behavioural arenas (Fig. 1) (e.g. from 14 to 8°C, activity *Q*_10_ values were 16,546±12,472 and 267±122 during daytime in the diurnal cunner and pumpkinseed, respectively, and 5.4±1.7 at night in the nocturnal mummichog; *Q*_10_ is unsuitable to the eels' proportional vigilance score). Activity is a highly flexible trait, even in the cold; alterations in activity can happen more quickly and with fewer physiological modifications than MRD. Thus, activity modulation may allow species to exit their dormant state more easily under stressful conditions (e.g. predation, hypoxia) or to exploit temporarily favourable environmental conditions (e.g. opportunistic foraging). Indeed, the reductions in activity during overwintering have been proposed to be facultative rather than obligate in many fish species (Kolok, 1991; Suski and Ridgway, 2009; Hasler et al., 2009). Lethargic or dormant fish species may simply prioritize activity reduction in the cold to minimize energy expenditure. This prioritization may lead to the evolution of a consistent adaptive response within species, such as the striking dormancy of cunner. In contrast, lethargic species retain flexibility in their overwintering behaviour possibly in response to variability in prey or predator presence, temperature and energy reserves (Micucci et al., 2003; Garvey et al., 2004). Indeed, Karchesky and Bennet (2004) showed individual variation in overwintering activity among largemouth bass: some remained inactive within an overwintering area, while others migrated freely between areas. The inherent flexibility of activity level makes it a convenient mechanism to conserve energy, or expend it as needed, in dynamic overwintering environments.

## Supplementary Material

Supplementary information
