# Supplementary material for: Inactivity and the passive slowing effect of cold on resting metabolism as the primary drivers of energy savings in overwintering fishes
Source: J Exp Biol. 2022 Apr 21;225(8):jeb243407. doi: 10.1242/jeb.243407 (PMC9124485; doi:10.1242/jeb.243407)
Supplement: Supplementary information [file jexbio-225-243407-s1.pdf]

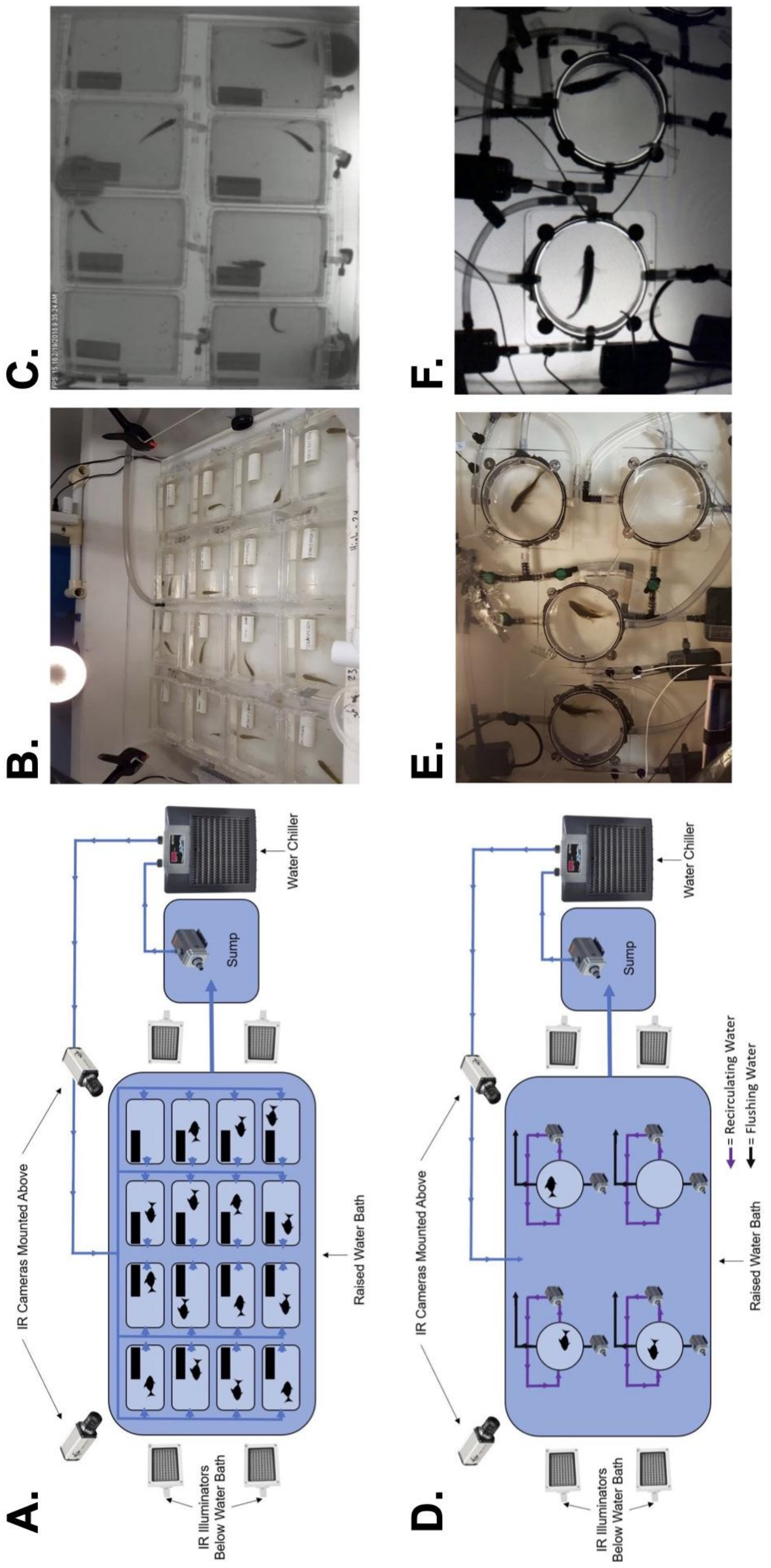

Journal of Experimental Biology • Supplementary information

Fig. S1. Schematics and representative camera images of the experimental systems used to measure behavioural responses to cooling in Experiment 1 (panels A, B, C) and to simultaneously measure fish oxygen consumption rate and spontaneous activity in Experiment 2 (panels D, E, F). In the schematics (A, D), blue arrows represent the direction of water flow pumped through the recirculating temperature-controlled system using the submersible pump in the sump. In the schematic for Experiment 2 (D), purple arrows represent the water flow recirculated within each respirometer (circles containing fish icon or empty circle for background respiration) by an in-line submersible pump, and black arrows represents the intermittent flow of flushing water to replenish the respirometers with oxygenated water using submersible flush pumps. Each respirometer was fitted with an oxygen optode and temperature probe (not shown) to measure oxygen consumption rate. IR, infrared. The camera images for Experiment 1 are a photograph showing mummichog (*Fundulus heteroclitus*) housed in their individual arenas (B) and a still image from an infrared video recording from above of cunner in their individual arenas (*Tautogolabrus adspersus*) (C). Both camera images were taken during the day when water temperatures were ~14°C. The camera images for Experiment 2 are a photograph of three pumpkinseed sunfish (*Lepomis gibbosus*) in their respirometers and an empty chamber to measure background respiration (E), and a still image from an infrared video recording of two of the fish (in total, two infrared-sensitive cameras were used) (F). See *Experiment 1: Behavioural Responses to Cooling to Winter Temperature* and *Experiment 2: The Effect of Acute Cooling and Cold Acclimation on Spontaneous Activity and Metabolic Rate, and their Relationship* in *Methods* in the main text for details of the specific equipment used. The size of the arenas in Experiment 1 (A-C), and thus the number of arenas within the system, varied based on species size (see Table S1).

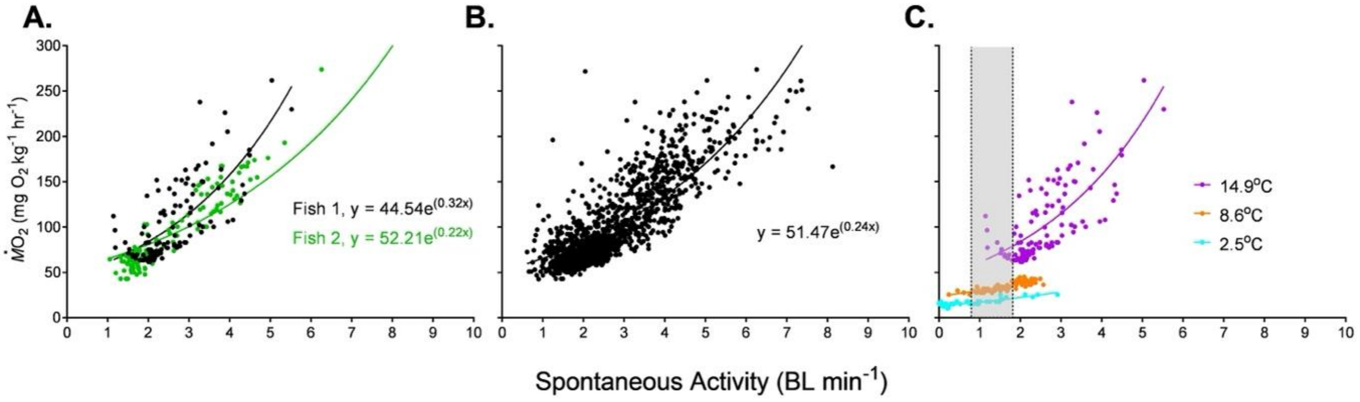

Fig. S2. Visual representations of the three methods we used to estimate standard metabolic rate (SMR) or otherwise control for activity variation across temperatures using the measured relationships between spontaneous activity and $\dot{M}O_2$ in the study species in Experiment 2. The data shown is representative data for pumpkinseed sunfish. Panel A shows Approach 1, our primary approach which was used to calculate the data shown in Figure 6 in the main text: estimating SMR of individual fish at each exposure temperature from the y-intercept of the relationship for that fish (the data for two representative individual fish at 14°C are shown in black and green; their SMR in this case are 44.54 and 52.21 mg O_2 kg^{-1} hr^{-1}). Panel B shows Approach 2: estimating group SMR from the y-intercept of the relationship for all individuals combined at each temperature (the data for 14°C is shown, the group SMR in this case is 51.47 mgO_2 kg^{-1} hr^{-1}) (see Figure 5 in main text). Panel C shows Approach 3: calculating the activity-controlled metabolic rate by averaging the metabolic rate within each exposure temperature across a similar range of activity (0.8 – 1.8 BL min^{-1}, shown as a grey bar bounded by dashed lines) (this example would result in three activity-controlled metabolic rate values, one for each temperature). For more details on each method see main text, *Experiment 2: Estimating Thermal Sensitivities (Q_{10}) of Standard Metabolic Rate (SMR) and Metabolic Rate at a Standardized Activity Level* in *Data Analysis and Statistics*.

Supplementary Methods: Experiment 2: Acute Thermal Sensitivity of Routine Metabolic Rate

For comparison to our analyses of metabolic rate where variation in activity was controlled for (i.e., SMR and activity-controlled metabolic rate), the thermal sensitivity of routine metabolic rate (RMR) was investigated. First, the thermal sensitivity of group RMR was calculated by exponentially relating all temperature recordings during the acute cooling period with the corresponding

measurements of $\dot{M}O_2$ from all fish of a given species in Experiment 2. The equation of the exponential relationship between temperature and $\dot{M}O_2$ for each species was used to calculate a single species-specific group Q_{10} for routine $\dot{M}O_2$ across the acute cooling temperatures, as follows: $Q_{10} = e^{(slope \cdot 10)}$, where e is the natural exponent, slope is the slope of the relationship, and 10 represents a 10°C increment. Second, for each species, every individual fish's RMR was calculated at each experimental temperature by averaging all measurements of $\dot{M}O_2$ for the individual at that temperature. The individual RMR values were averaged to determine mean RMR at each temperature for each species and then were then used to individually calculate thermal sensitivity of RMR in response to acute cooling, acclimation to ~2.5^{o}C for 4-6 weeks, and acute rewarming to their holding temperature.

Journal of Experimental Biology • Supplementary information

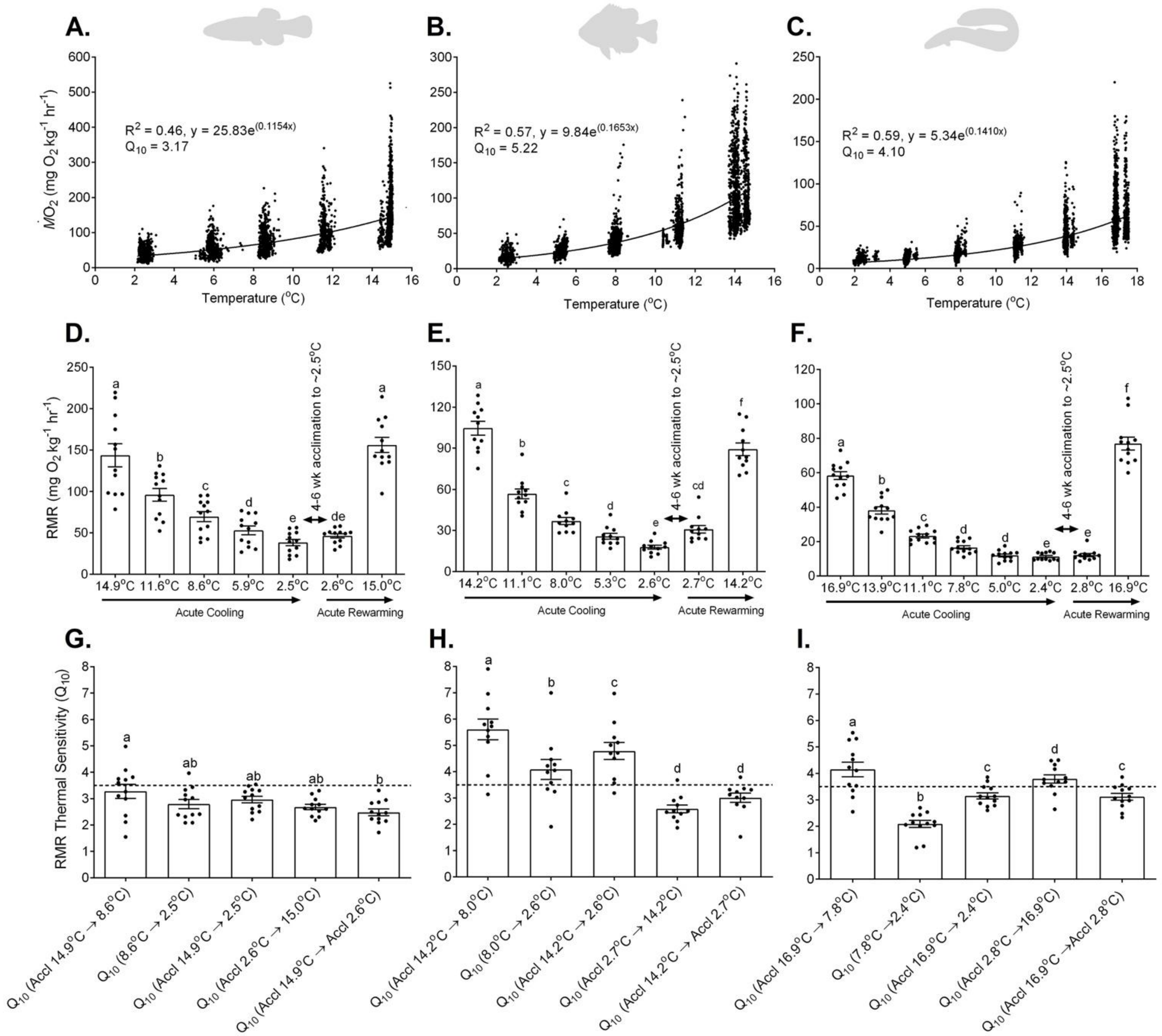

Fig. S3. The effect of acute cooling and cold acclimation followed by rewarming on routine metabolic rates of mummichog, pumpkinseed sunfish, and American eel (Experiment 2). Panels A-C show the effect of acute cooling on metabolic rate (oxygen consumption rate, $\dot{M}O_2$) in all measurement intervals for all individuals in mummichog (A) (n = 12), pumpkinseed sunfish (B) (n = 11), and American eel (C) (n = 12). The data were fitted with an exponential regression and a Q_{10} value across all exposure temperatures without controlling for variation in spontaneous activity was calculated as $Q_{10} = e^{(slope \cdot 10)}$. All regressions were highly significant (p<0.0001; df = 4644, 4023, and 5425 for mummichog, pumpkinseed, and American eel, respectively). Panels D-F show the routine metabolic rates (RMR) in mummichog (D), pumpkinseed (E), and American eel (F) during acute cooling from an initial warm

Journal of Experimental Biology • Supplementary information

acclimation ("Accl") temperature, 4-6 weeks acclimation to cold (~2.5^{o}C), and acute rewarming of cold-acclimated fish. RMR were calculated by averaging all $\dot{M}O_2$ measurements for each individual fish at each exposure temperature (i.e. RMR are derived from the data shown in panels A-C). Panels G-I show the thermal sensitivities (Q_{10}) of RMR calculated using the individual fish's RMR values shown in Panels D-F across several intervals relating to the temperature exposures in Experiment 2 (these Q_{10} values correspond to the "Average $\dot{M}O_2$" Q_{10} values in Table S3). For panels D-I, data are means ± s.e.m. (n=12, 11, and 12, for mummichog, pumpkinseed, and American eel respectively) with overlying black circles representing the individual RMR values or Q_{10} values. The dotted horizontal line represents our defined Q_{10} threshold that infers metabolic rate depression, when applied to SMR (i.e., $Q_{10} > 3.5$). Values with different letters are significantly different (linear mixed effects models and Bonferroni post-hoc multiple comparisons tests, $p<0.05$).

Journal of Experimental Biology • Supplementary information

Table S1. Parameters for study animals and behavioural arenas or respirometry in Experiment 1 and 2.

	Species	N	Fish Total Length (cm)	Fish Weight (g)	Arena Dimensions (cm)	Arena Water Depth (cm)	Shelter Size (Length x Internal Diameter, Material)	Respirometer Volume, Dimensions, Material	Respirometer Closed Period (temperature, time in min)
**Experiment 1**	Cunner	16	6.4 ± 0.1	5.1 ± 0.3	20.2 x 15.6 x 9.7	~8	8 cm x 1.9cm, PVC	NA	NA
	Mummichog	16	6.6 ± 0.2	5.7 ± 0.4	20.2 x 15.6 x 9.7	~8	8 cm x 1.9cm, PVC	NA	NA
	Pumpkinseed	12	11.8 ± 0.2	30.0 ± 0.7	38.3 x 24.1 x 14.1	~11	15 cm x 1.9cm, PVC	NA	NA
	American Eel	12	9.8 ± 0.2	1.1 ± 0.1	20.2 x 15.6 x 9.7	~5	10 cm x 1.3cm, PVC	NA	NA
**Experiment 2**	Mummichog	12	9.1 ± 0.2	Pre: 8.7 ± 0.5<br>Post: 8.3 ± 0.5	NA	NA	NA	252.4 ± 8.8 ml, circular, 10.1 cm i.d. x 2.5 cm i.h., acrylic	14°C, 15; 11°C, 15; 8°C, 25; 5°C, 40; 2°C, 55
	Pumpkinseed	11	12.3 ± 0.1	Pre: 28.5 ± 1.2<br>Post: 26.8 ± 1.0	NA	NA	NA	1496.7 ± 21.0 ml, circular, 16.5 cm i.d. x 6.4 cm i.h., acrylic	14°C, 15; 11°C, 15; 8°C, 25; 5°C, 40; 2°C, 55
	American Eel	12	NM	Pre: 4.1 ± 0.2<br>Post: 3.7 ± 0.2	NA	NA	NA	145.5 ± 3.5 ml, rectangular, 6 cm i.w. x 6 cm i.l. x 5 cm i.h., glass	17°C, 10; 14°C, 15; 11°C, 25; 8°C, 40; 5°C, 55; 2°C, 70

Data are means ± s.e.m. except respirometer dimensions where data are means ± s.d. The flush period for respirometry was always 5 minutes. NM, not measured. NA, not applicable. i.d., i.h., i.w., and i.l. represent internal diameter, internal height, internal width, and internal length, respectively. Pre- and post- weights in Experiment 2 were measured at the beginning of the acute cooling trial (i.e. in warm-acclimated fish) and after 4-6 weeks of acclimation to ~2.5°C followed by acute rewarming, respectively.

Table S2. (See next page) Summary of statistical outputs obtained from GLMMs examining the effects of diel cycle (Day vs. Night) and acute cooling on spontaneous activity, sheltering, food consumption, and vigilance (in eels only) of four species of putatively winter-dormant fishes. These analyses correspond to the data shown in Fig. 1 in the main text. Significant effects were calculated using type II Wald chi-square tests and ($p < 0.05$) are indicated in bold.

Independent Factor	Dependent Factor	Cunner			Pumpkinseed Sunfish			Mummichog			American Eel		
		**Chisq**	**df**	**p**	**Chisq**	**df**	**p**	**Chisq**	**df**	**p**	**Chisq**	**df**	**p**
Spontaneous Activity	Temperature	3600.68	14	**<0.0001**	2490.64	14	**<0.0001**	312.520	15	**<0.0001**	NA		
	Day/Night	338.33	1	**<0.0001**	272.03	1	**<0.0001**	54.517	1	**<0.0001**			
	Interaction	2311.39	14	**<0.0001**	1132.40	14	**<0.0001**	16.412	14	0.2889			
Food Consumption	Temperature	377.14	14	**<0.0001**	682.52	14	**<0.0001**	860.89	15	<0.0001	668.46	17	**<0.0001**
Sheltering	Temperature	159.0956	14	**<0.0001**	272.743	14	**<0.0001**	61.9898	15	**<0.0001**	NA		
	Day/Night	3.1676	1	0.0751	21.794	1	**<0.0001**	1108.845	1	**<0.0001**			
	Interaction	47.8360	14	**<0.0001**	222.444	14	**<0.0001**	6.1437	14	0.9627			
Vigilance	Temperature	NA			NA			NA			224.203	17	**<0.0001**
	Day/Night										418.734	1	**<0.0001**
	Interaction										43.641	14	**0.0004**

Chisq is the chi-squared test-statstic and df are the degrees of freedom. NA, not applicable.

Table S3 (See next page) Thermal sensitivity quotients (Q_{10}) of SMR of mummichog (n = 12), pumpkinseed (n=11), and American eel (n=12) using different methods of estimating SMR, as well as Q_{10} for activity-controlled metabolic rate ("$Q_{10\ (Overlapping\ SA)}$") and routine metabolic rate ("$Q_{10\ (Average\ \dot{M}O2)}$") (Experiment 2). "$Q_{10\ (Ind\ Extrapolated)}$" and "$Q_{10\ (Grp\ Extrapolated)}$" refer to the Q_{10}'s calculated using SMR values estimated by extrapolating $\dot{M}O_2$ to zero activity in individuals (i.e. Approach 1 described in Methods of main text; see Fig. S2A; values also reported in Fig. 6) or across all experimental fish (i.e. Approach 2 described in Methods of main text; see Fig. S2B; values also reported in Fig. 5), respectively. "$Q_{10\ (Overlapping\ SA)}$" refers to Q_{10}'s calculated using the average of each individual's $\dot{M}O_2$ values within a common, overlapping range of spontaneous activity (SA) across all temperatures (i.e. Approach 3, "Activity-controlled metabolic rate", described in Methods of main text; see Fig. S2C). Due to differing overlapping ranges of SA, the Q_{10} between acclimated 14.9°C and acclimated 2.6°C could not be calculated for mummichogs (see Methods in main text). "$Q_{10\ (Lowest\ 20\ \dot{M}O2)}$" and "$Q_{10\ (Lowest\ 20\ SA)}$" refer to the Q_{10}'s calculated using the average of each individual's lowest 20 $\dot{M}O_2$ points at each temperature and by using the average $\dot{M}O_2$ associated with the lowest 20 SA measurements at each temperature, respectively. "$Q_{10\ (\ Extrapolated\ +\ \dot{M}O2)}$" and "$Q_{10\ (Extrapolated\ +\ SA)}$" refer to the Q_{10}'s calculated using SMR values estimated by extrapolating $\dot{M}O_2$ to zero activity in individual fish and, where this relationship was not significant, replacing the extrapolated SMR value with the value of the average of the lowest 20 $\dot{M}O_2$ points or with the value for average $\dot{M}O_2$ associated with the lowest 20 SA measurements, respectively, and in the same individual fish. Note that "$Q_{10\ (Extrapolated\ +\ \dot{M}O2)}$" and "$Q_{10\ (Extrapolated\ +\ SA)}$" are not included for American eel as all relationships within individuals were significant. "$Q_{10\ (Average\ \dot{M}O2)}$" refers to the Q_{10} calculated using the average of all $\dot{M}O_2$ values for each individual fish at each temperature (i.e. routine $\dot{M}O_2$, or routine metabolic rate), and is also reported in Fig. S3G-I. Q_{10}'s above our defined threshold for metabolic rate depression (i.e. Q_{10} >3.5, for SMR) are bolded. The Q_{10} values of different temperature intervals within each Q_{10} row were compared with linear mixed effects models; italicized P-values indicate there is a significant effect of temperature interval on Q_{10} values ($p < 0.05$). Where a significant effect was observed, values that share letters are not significantly different (Bonferroni post-hoc multiple comparisons tests, $p < 0.05$).

Journal of Experimental Biology • Supplementary information

Species	Temperature Interval	Q_{10} (Ind Extrapolated)	Q_{10} (Grp Extrapolated)	Q_{10} (Overlapping SA)	Q_{10} (Lowest 20 $\dot{M}O_2$)	Q_{10} (Lowest 20 SA)	Q_{10} (Extrapolated + $\dot{M}O_2$)	Q_{10} (Extrapolated + SA)	Q_{10} (Average $\dot{M}O_2$)
Mummichog	**Accl 14.9°C → 8.6°C**	3.17 ± 0.17	2.65	2.83 ± 0.20	3.24 ± 0.27	3.17 ± 0.26[acd]	3.17 ± 0.17	3.17 ± 0.18	3.28 ± 0.27[a]
	**8.6°C → 2.5°C**	3.04 ± 0.28	3.28	2.55 ± 0.25	2.59 ± 0.17	2.57 ± 0.19[b]	3.02 ± 0.29	2.92 ± 0.32	2.79 ± 0.17[ab]
	**Accl 14.9°C → 2.5°C**	3.05 ± 0.12	2.93	2.64 ± 0.20	2.87 ± 0.15	2.82 ± 0.15[abd]	3.04 ± 0.13	2.97 ± 0.13	2.97 ± 0.13[ab]
	**Accl 2.6°C → 15.0°C**	3.16 ± 0.19	2.9	3.26 ± 0.13	**3.52 ± 0.18**	3.15 ± 0.14[cd]	3.14 ± 0.19	3.18 ± 0.20	2.68 ± 0.10[ab]
	**Accl 14.9°C → Accl 2.6°C**	3.19 ± 0.24	2.42	NA	3.24 ± 0.23	2.96 ± 0.20[d]	3.19 ± 0.24	3.19 ± 0.19	2.48 ± 0.13[b]
	**P-Value**	0.97	NA	0.612	0.1189	*0.0053*	0.9897	0.4967	*0.0181*
Pumpkinseed	**Accl 14.2°C → 8.0°C**	3.27 ± 0.47	3.08	3.25 ± 0.18	**3.97 ± 0.36[a]**	**3.69 ± 0.34[a]**	3.36 ± 0.46	3.18 ± 0.50	**5.61 ± 0.40[a]**
	**8.0°C → 2.6°C**	2.40 ± 0.30	3.19	3.13 ± 0.37	**3.95 ± 0.51[a]**	**3.99 ± 0.34[a]**	2.92± 0.51	2.72 ± 0.38	**4.08 ± 0.38[b]**
	**Accl 14.2°C → 2.6°C**	2.67 ± 0.21	3.13	3.10 ± 0.17	**3.77 ± 0.23[a]**	**3.74 ± 0.21[a]**	2.93 ± 0.29	2.70 ± 0.21	**4.79 ± 0.33[c]**
	**Accl 2.7°C → 14.2°C**	2.96 ± 0.25	2.93	2.68 ± 0.14	2.89 ± 0.17[b]	2.75 ± 0.20[b]	2.92 ± 0.24	2.89 ± 0.25	2.59 ± 0.15[d]
	**Accl 14.2°C → Accl 2.7°C**	2.30 ± 0.26	2.45	NA	2.61 ± 0.16[b]	2.49 ± 0.16[b]	2.29 ± 0.26	2.28 ± 0.27	3.01 ± 0.17[d]
	**P-Value**	0.0712	NA	0.2595	*0.0041*	*<0.0001*	0.2399	0.341	*<0.0001*
American Eel	**Accl 16.9°C → 7.8°C**	**3.73 ± 0.23[ad]**	**3.53**	**3.57± 0.21[ad]**	3.47 ± 0.24[ad]	**3.81 ± 0.27[ad]**	NA	NA	**4.15 ± 0.28[ad]**
	**7.8°C → 2.4°C**	1.99 ± 0.10[b]	1.94	1.89 ± 0.08[b]	1.74 ± 0.11[b]	1.95 ± 0.10[b]	NA	NA	2.09 ± 0.13[b]
	**Accl 16.9°C → 2.4°C**	2.91 ± 0.09[c]	2.82	2.78 ± 0.07[c]	2.62 ± 0.08[c]	2.92 ± 0.12[c]	NA	NA	3.15 ± 0.11[c]
	**Accl 2.8°C → 16.9°C**	**3.61 ± 0.10[d]**	3.42	**3.51 ± 0.08[d]**	**3.54 ± 0.12[d]**	**3.73 ± 0.05[d]**	NA	NA	**3.79 ± 0.16[d]**
	**Accl 16.9°C → Accl 2.8°C**	2.90 ± 0.08[c]	2.78	2.85 ± 0.08[c]	2.86 ± 0.07[c]	2.98 ± 0.08[c]	NA	NA	3.11 ± 0.13[c]
	**P-Value**	*<0.0001*	NA	*<0.0001*	*<0.0001*	*<0.0001*	NA	NA	*<0.0001*

Data are means ± s.e.m. NA, not applicable. Accl, acclimated (all other temperatures are acute exposures).
